# Tectonic setting shapes microbial biosynthetic potential across global geothermal environments

**DOI:** 10.1101/2025.09.14.675129

**Published:** 2026-04-30

**Authors:** Ana Clara Pelliciari Silva, Benoit de Pins, Francesco Montemagno, Flavia Migliaccio, Martina Cascone, Deborah Bastoni, Bernardo Barosa, Matteo Selci, Costantino Vetriani, Agostina Chiodi, Federico A. Vignale, Maria Garcia Alai, Alberto Vitale Brovarone, Gerdhard L. Jessen, Jenny M. Blamey, J. Maarten de Moor, Karen G. Lloyd, Peter Barry, The CoEvolve Project consortium, Donato Giovannelli

**Affiliations:** 1- Department of Biology, University of Naples “Federico II”, Naples, Italy; 2- National Biodiversity Future Center, Palermo, Italy; 3- Department of Marine and Coastal Science, Rutgers University, New Brunswick, NJ, USA; 4- Department of Biochemistry and Microbiology, Rutgers University, New Brunswick, NJ, USA; 5- Instituto de Bio y Geociencias del NOA (IBIGEO, UNSa-CONICET), Av. 9 de Julio 14, A4405BBA Salta, Argentina; 6- European Molecular Biology Laboratory - Hamburg Unit, Hamburg, Germany; 7- Department of Biology and Geology, BIGEA, University of Bologna “Alma Mater Studiorum”, Bologna, Italy; 8- CNRS; 9- Instituto de Ciencias Marinas y Limnológicas, Universidad Austral de Chile, Valdivia, Chile & Centro de Investigación Oceanográfica COPAS COASTAL, Universidad de Concepción, Chile; 10- Fundación Biociencia & Universidad de Santiago de Chile; 11- Observatorio Vulcanológico y Sismológico de Costa Rica, Universidad Nacional, Heredia, Costa Rica; 12- Department of Earth and Planetary Sciences, University of New Mexico, Albuquerque, NM, USA; 13- Earth Science Department, University of Southern California, Los Angeles, CA, USA; 14- Marine Chemistry & Geochemistry Department, Woods Hole Oceanographic Institution; 15- National Research Council - Institute of Marine Biological Resources and Biotechnologies - CNR-IRBIM, Ancona, Italy; 16- Earth-Life Science Institute, Tokyo Institute of Technology, Tokyo, Japan

**Keywords:** biosynthetic gene clusters, geothermal systems, extreme environments, extremophiles, terpenes

## Abstract

Microbial communities in geothermal environments constitute an underexplored reservoir of biosynthetic gene clusters with significant biotechnological potential. Here, we investigate the secondary metabolite potential of 219 microbial communities across marine and continental geothermal field sites, encompassing broad environmental gradients in temperature (4.7 °C to 93.5 °C), pH (0.85 to 10.3), and tectonic setting, including convergent margins, divergent margins at mid-ocean ridges, and paleo-convergent intraplate plume systems. We identified 9,019 putative biosynthetic gene cluster families, mostly lacking similarity to known biosynthetic gene clusters. Volcanic arc systems consistently exhibited the highest diversity of biosynthetic repertoires, whereas intraplate plume systems showed a greater representation of terpene-associated gene cluster families. In contrast, divergent margin systems were primarily characterized by nonribosomal peptide synthetase and ribosomally synthesized and post-translationally modified peptide pathways, together accounting for a large fraction of their predicted biosynthetic diversity. These findings suggest that tectonic context could be associated with large-scale patterns in microbial biosynthetic potential and provide a geobiological framework for guiding future natural product discovery in geothermal environments.

## Introduction

Extreme environments are increasingly recognized as reservoirs of biosynthetic gene clusters (BGCs) and potentially novel chemistries^1,2^. Across deserts ^1^, deep-sea systems^3^, polar soils^4^, and hypersaline habitats^5^, genome mining and cultivation studies have revealed extensive unexplored biosynthetic diversity, including in microbial lineages historically underexplored for secondary metabolism^6^. Prior work has not only stated geothermal extremophiles as an untapped source of natural products^7,8^, but also highlighted that environmental stressors often correlate with enrichment of specific BGC classes in the microbiome: for example, chronic metal exposure favors NRPS-linked (nonribosomal peptide synthetases) metallophores that detoxify metals^9,10^, whereas extremely acidic, heat-stressed systems dominated by few chemolithotrophs often show a dearth of large NRPS/PKS (polyketide synthases) clusters^11,12^. These patterns might indicate that BGC repertoires reflect the selective pressures of their environment. It is important to notice, however, that the geochemical landscapes in which microbial communities adapt to are not random but are firmly rooted in the geological settings from which they arise^13^. The mineral content, redox potential, temperature and ionic composition of each environment are shaped by underlying tectonic and geological processes, which in turn influence microbial community structure and function^13,14^.

Genome and metagenome mining shows that most BGCs in microbes encode unknown products, revealing a huge reservoir of undiscovered natural compounds with potential as antibiotics, anticancer agents, and agrochemicals^15–17^. Global ocean microbiomes alone harbor ~40,000 mostly new BGCs^15^. Similar untapped BGC richness is found in *Bacillus*, *Streptomyces* and Antarctic Actinomycetes, where 80–90% of clusters are unknown^16^. Using these approaches, localised settings have also been explored such as microbial mats of Shark Bay^5^, glacier-fed stream biofilms^18^, or the Tibetan Plateau extreme aquatic microbiomes^19^. Nevertheless, despite the long-standing biotechnological relevance of geothermal systems^20,21^, large-scale surveys of their biosynthetic potential remain limited.

Here, we address this knowledge gap by investigating the putative biosynthetic potential of global geothermal extreme environments. We combine comprehensive analyses of 219 microbial communities from marine and continental geothermal sites to map the diversity and distribution of predicted BGCs from distinct gene cluster families (GCFs) across diverse tectonic settings. Our results show that variation in tectonics and geochemical context might be associated with distinct patterns in the composition and diversity of predicted secondary metabolite biosynthetic repertoires. Together, these findings provide a global framework for understanding how geological and environmental gradients can relate with microbial biosynthetic potential and highlight geothermal systems as reservoirs of unexplored genomic diversity relevant for future bioprospecting efforts.

## Results

**Figure 1-:**
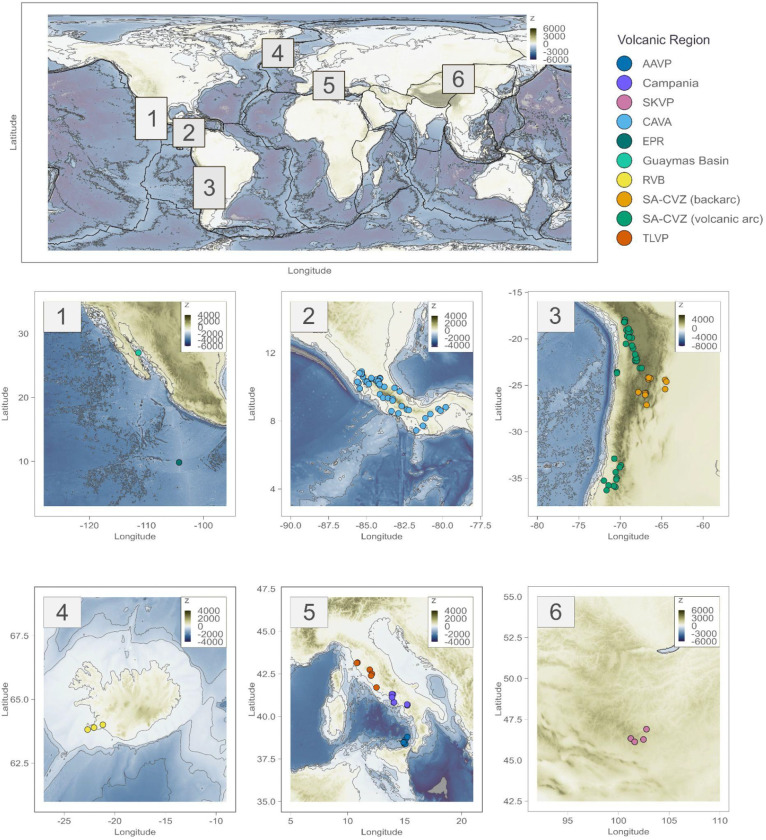
Geographic distribution of sampled geothermal extreme environments included in this study. Locations span diverse volcanic provinces in the Central America Volcanic Arc (CAVA), the Guaymas basin, the East Pacific Rise (EPR), the South American Central Volcanic Zone (SA-CVZ), the Reykjanes Volcanic Belt (RVB), the South Khangai Volcanic Province (SKVP) and the Tuscan-Latium Volcanic Province (TLVP), the Campania region, and the Aeolian Arc Volcanic Province (AAVP).

We analyzed 219 microbial communities from geothermal features across ten major volcanic provinces using a combination of metagenomic sequencing and geochemical measurements (Figure 1). These include environments in present-day convergent margins, encompassing volcanic arcs (*e.g.*, the South American Central Volcanic Zone (SA-CVZ), Central America Volcanic Arc (CAVA), and Aeolian Arc Volcanic Province (AAVP)), adjacent back-arc regions (*e.g.,* part of the SA-CVZ), and transitional post-subduction extensional arcs (Campania, southern Italy and the Tuscan-Latium Volcanic Province, (TLVP) in central Italy); plume-influenced mid-ocean ridge settings (Reykjanes Volcanic Belt, RVB); intracontinental magmatic systems in paleoaccretionary environments in the South Khangai Volcanic Province (SKVP); and deep-sea vents in the Guaymas basin and the East Pacific Rise (EPR) (Figure 1).

**Figure 2-:**
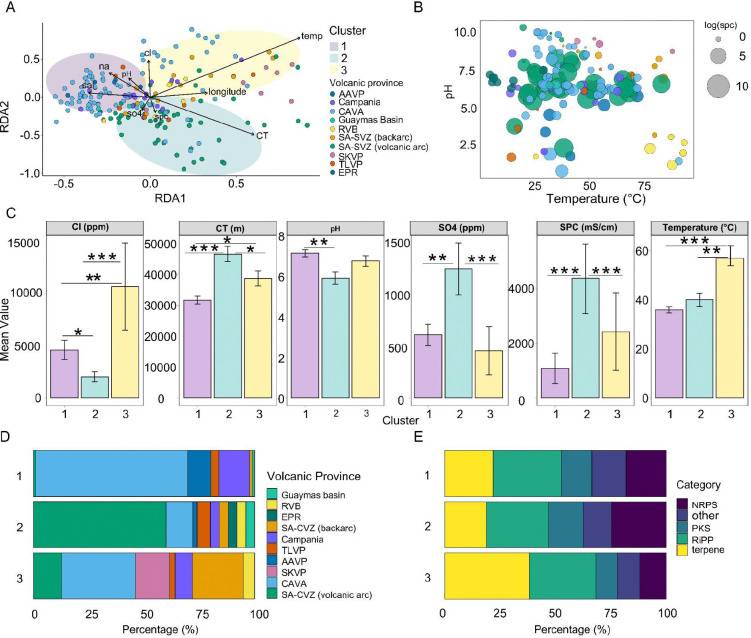
Biosynthetic gene cluster family (GCF) composition across geothermal sites. **(a)** Partial redundancy analysis (RDA) ordination of GCF composition across geothermal metagenomes. Cluster 1: majorly composed of subduction-driven volcanic arc geothermal sites, Cluster 2: subduction-driven volcanic arc and a strong presence of sites present in divergent tectonics such as MOR (Mid-Ocean Ridges). Cluster 3: diverse tectonic settings with abundant samples in backarc tectonics, volcanic arcs and intracontinental plumes. Points are colored by volcanic province of origin: CAVA (Central American Volcanic Arc), SA-CVZ (South American Central Volcanic Zone), RVB (Reykjanes Volcanic Belt), TLVP (Tuscan-Latium Volcanic Province), AAVP (Aerolian Arc Volcanic Province), Campania region, SKVP (South Khangai Volcanic Province), the Guaymas basin, and the EPR (East Pacific Rise). Arrows indicate significant environmental variables, with direction and length proportional to their correlation with ordination axes (*e.g.*, pH, temperature, salinity, conductivity (spc), crustal thickness (CT), Na content, Cl^-^ content, SO_4_ content and longitude). Colored convex hulls illustrate the spatial distribution of three k-means clusters, used here to highlight compositional similarities. **(b)** pH as a function of temperature of samples. Dots size correlated with the log conductivity values (spc) of the samples, and coloured by volcanic province of origin. **(c)** Environmental variables by cluster. Mean ± standard error (SE) values are shown for six key environmental parameters across microbial community clusters (1–3). Colors represent cluster identity: pale purple (Cluster 1), pale teal (Cluster 2), and pale yellow (Cluster 3). Statistical differences were tested using the Kruskal-Wallis test followed by Dunn’s post-hoc comparisons with Benjamini-Hochberg correction. Asterisks indicate significant pairwise differences between clusters (*p < 0.05, **p < 0.01, ***p < 0.001). **(d)** Volcanic province origin across tectonic clusters. Stacked horizontal bar plots show the proportion of each volcanic province represented within each k-means-derived cluster. **(e)** Relative distribution of major GCF classes across sample clusters reflecting presence/absence of biosynthetic profiles. Cluster 1 (volcanic arc majorly) exhibits a more even distribution across GCF classes. Cluster 2 (MOR at divergent tectonic settings and volcanic arc at convergent settings majorly) shows a higher relative proportion of NRPS (nonribosomal peptide synthetases). Cluster 3 (rich in backarc and intracontinental plume samples) contains higher contributions from terpenes.

Biosynthetic potential was characterized using presence–absence profiles of gene cluster families (GCFs). Partial redundancy analysis (RDA) revealed a structured distribution of samples in ordination space associated with environmental gradients (overall model: F = 1.69, p < 0.001; Figure 2). Clustering of RDA site scores identified three major groups of samples. These clusters differed significantly in biosynthetic composition (PERMANOVA on Hellinger–Euclidean distances: R^2^ = 0.039, p < 0.001; ANOSIM R = 0.26, p < 0.001). Samples originating from volcanic arc, back-arc, and mid-ocean ridge systems occupied partially distinct regions of ordination space, suggesting that geothermal environments associated with different tectonic contexts might be characterized by distinct combinations of biosynthetic gene cluster families (Figure 2). Sample clusters 1 and 2 were both dominated by volcanic arc systems (> 80% of samples), but differed in environmental associations: Cluster 1 was linked to higher pH, whereas Cluster 2 was associated with greater crustal thickness. In contrast, Cluster 3 included a stronger contribution from back-arc and intracontinental plume settings and aligned with higher temperatures.

We next examined biosynthetic composition across clusters. NRPS-associated GCFs are most frequent in Cluster 2 (26%), followed by Cluster 1 (19%) and Cluster 3 (12%). In contrast, terpene-associated GCFs have higher representation in Cluster 3 (39%) relative to Clusters 1 (21%) and 2 (18%). RiPP-associated GCFs are broadly distributed across all clusters (28–31%), whereas PKS-associated GCFs are less frequent overall (9–15%). Remaining GCFs, including hybrid and unclassified clusters, comprised a smaller fraction of the biosynthetic repertoire.

Among the environmental variables tested, temperature, pH, and crustal thickness showed consistent associations with variation in biosynthetic GCF composition. Marginal PERMANOVA analyses revealed that temperature explained the largest proportion of variance (R^2^ = 0.012, F = 2.50, p < 0.001), followed by pH (R^2^ = 0.010, F = 2.07, p < 0.001) and crustal thickness (CT; R^2^ = 0.007, F = 1.33, p < 0.01). Although each variable individually accounted for a modest fraction of total variance, their combined effects define a multidimensional environmental gradient that might have an influence on biosynthetic repertoires across geothermal systems (Figure S1). Notably, these variables can covary across tectonic environments, which can reflect linked geological processes that influence fluid chemistry and thermal regimes. When analysing samples in active convergent margins, volcanic arc geothermal systems exhibit greater putative GCF diversity in comparison to backarc and post-subduction extensional arcs (volcanic arc: 2.18 ± 0.69; backarc: 1.85 ± 0.57; post-subduction extensional arcs: 1.88 ± 0.41; Kruskal–Wallis χ^2^ = 17.35, adjusted p = 1.7 × 10⁻⁴, Figure S2).

To evaluate whether differences in GCF composition could be explained by underlying microbial community structure, we characterized taxonomic composition from assembled contigs and performed variation partitioning to quantify the relative contributions of environmental variables and taxonomic composition to variation in GCF repertoires. Environmental predictors independently explained a larger fraction of biosynthetic variation (adjusted R^2^ = 0.058) than taxonomy alone (adjusted R^2^ = 0.046), while a smaller shared fraction was attributable to covariation between environment and taxonomy (adjusted R^2^ = 0.017) (Figure S3). Both environmental and taxonomic fractions were statistically significant (permutation tests, p = 0.001), indicating that biosynthetic composition reflects contributions from both environmental context and community structure.

**Figure 3 -:**
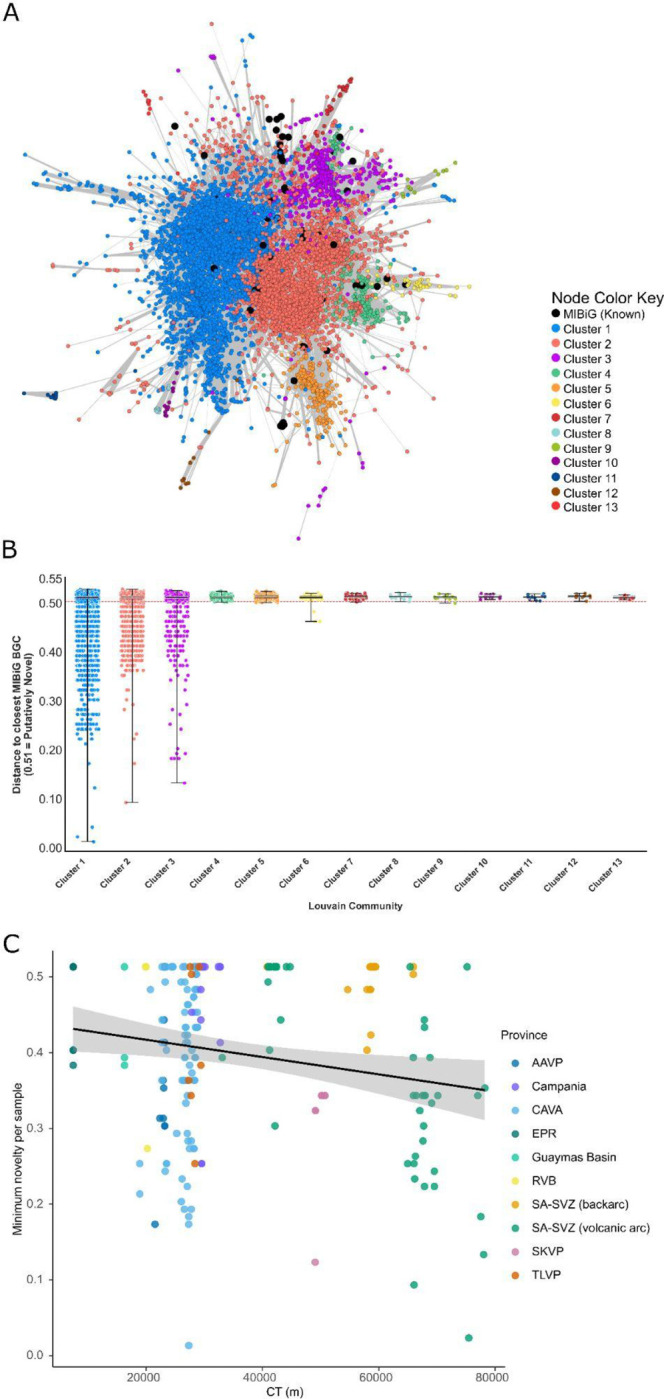
Network structure and novelty of putative terpene biosynthetic gene clusters. **(a)** Similarity network of predicted terpene biosynthetic gene clusters generated using BiG-SCAPE (Biosynthetic Gene Similarity Clustering and Prospecting Engine) at a distance cutoff of 0.5. Nodes represent BGCs (biosynthetic gene clusters) and edges indicate pairwise similarity (distance ≤ 0.5). Nodes are colored according to Louvain communities, and experimentally characterized reference clusters from the MIBiG (Minimum Information about a Biosynthetic Gene) are shown in black. Connected components correspond to predicted gene cluster families (GCFs). **(b)** Distribution of distances from each putative BGC to its closest MIBiG reference cluster, grouped by Louvain community. Each point represents a single predicted BGC, with boxplots summarizing the distribution within each community. Distances of 0.51 (above the cutoff, indicated by the dashed red line at 0.5) correspond to putative BGCs with no detectable similarity to any MIBiG cluster within the threshold and are therefore considered putatively novel. Communities with higher median distances and a greater proportion of values at 0.51 represent more putatively novel terpene BGC families. **(c)** Minimum terpene BGC novelty per sample as a function of crustal thickness (CT), colored by volcanic province. Each point represents a single sample, and the solid line indicates a linear regression with 95% confidence interval (grey shading). A weak but significant negative relationship is observed (R^2^ = 0.033, p = 0.0107), indicating a shift from more putatively novel BGCs in thin-crust settings (e.g., mid-ocean ridge systems) toward less novel, more reference-associated predicted BGCs in thicker crust environments typical of volcanic arcs.

While these analyses describe how putative biosynthetic gene cluster repertoires vary across environmental gradients, community structure and tectonic contexts, they do not address the extent to which this diversity might correspond to previously characterized biosynthetic pathways. We therefore next evaluated the novelty of predicted biosynthetic gene clusters by comparing the putative environmental BGCs to experimentally validated reference clusters. Network-based dereplication identified 9,019 GCFs across all samples (Supplementary Table 1, Figure 3 and Supplementary Figures S4-S6). A substantial fraction of these families show no network connections to known reference BGCs, indicating extensive unexplored biosynthetic diversity within geothermal microbiomes, an example can be seen in Figure 3. Analysis of the major biosynthetic classes revealed consistently high levels of novelty: 89% of terpene, 91% of NRPS, 94% of RiPP, and 97% of PKS GCFs (Supplementary Figures S4-S6) were not connected to experimentally characterized biosynthetic gene clusters. To further assess how novelty varies across environmental gradients, we evaluated the minimum novelty per sample for each major biosynthetic class. Across all classes, relationships with temperature and pH are weak and generally not statistically significant (R^2^ < 0.02; p > 0.05 in most cases, Supplementary Figures S7). In contrast, crustal thickness (CT) showed a consistent, albeit modest, negative relationship with minimum novelty for multiple classes, including terpene (R^2^ = 0.033, p = 0.0107), NRPS (R^2^ = 0.043, p = 0.0063), PKS (R^2^ = 0.041, p = 0.0070), and RiPP (R^2^ = 0.050, p = 0.0013) (Figure 3c, Supplementary Figures S4c–S5c). These results indicate that while overall novelty remains high across geothermal environments, large-scale geodynamic context, as approximated by crustal thickness, may exert a consistent influence on the degree of novelty amongst predicted environmental BGC. Notably, samples from mid-ocean ridge systems (EPR and Guaymas basin) tend to occupy the more novel end of this gradient, whereas subduction-related volcanic arc systems (convergent margins in the SA-CVZ) are associated with predicted BGCs that are more closely related to experimentally characterized pathways. This pattern may also, in part, reflect underlying sampling biases in current reference databases, which are disproportionately composed of terrestrial and easily accessible environments.

## Discussion

### Geology, geochemistry and BGCs

**Figure 4-:**
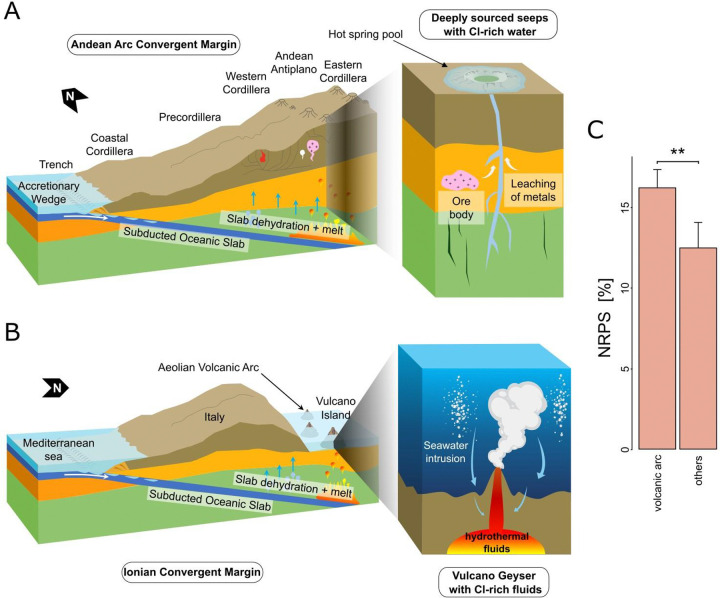
Schematic representation of volcanic arcs at convergent margins and their influence on microbial biosynthetic potential. (a) Schematic representation of the SA-CVZ (South American Central Volcanic Zone) volcanic arc and its influence on microbial biosynthetic potential. On the macro scale, the steeply dipping oceanic slab dehydrates, releasing Cl-enriched fluids from altered oceanic crust. These fluids migrate upward through faults and fractures in the continental crust. Metal leaching in these environments favor NRPS-rich (nonribosomal peptide synthetases) microbial communities. Macro-scale cross-section of the SA-CVZ adapted from ^24^. (b) Schematic of the Ionian convergent margin tectonic system and its role in shaping microbial biosynthetic potential in the AAVP (Aeolian Arc Volcanic Province). The subduction of the Ionian slab beneath the Aeolian Arc fuels arc magmatism and geothermal activity across southern Italy, and enhances mantle input in hydrothermal fluids. Beneath Vulcano Island, CO_2_ and chloride-bearing fluids ascend through permeable structures, mixing with meteoric water or seawater to form acidic, and metal-rich environments, selecting for a microbiome capable of withstanding heavy metal toxicity. Cross-section of the Vulcano Geyser adapted from ^117^. (c) At the microbiological level, volcanic arc microbiomes carry a predicted biosynthetic gene cluster repertoire potentially rich in NRPS, that support survival through metal chelation and detoxification. The bar plots show the per-sample proportional representation of NRPS biosynthetic potential, computed as the number of NRPS gene cluster families (GCFs) detected in each sample divided by the total number of detected GCFs in that sample (expressed as %), comparing 125 volcanic arc samples versus 87 samples from other active plate-tectonic settings. Differences between the two groups (“volcanic arc” vs “others”) were evaluated with the non-parametric two-sided Wilcoxon rank-sum (Mann–Whitney U) test. Significance is coded as p < 0.01 (**).

The extreme conditions and metal bioavailability in geothermal systems of active plate tectonic sites not only select which microbes thrive^22^, but also the BGC repertoire those communities harbor. Consistent with this ecological framework, our analyses reveal that variation in biosynthetic GCF composition across global geothermal systems is associated with both environmental context and underlying taxonomic structure (Figure S3). In our study, geothermal systems of volcanic arcs in subduction zones exhibit high potential for microbial BGC richness (Cluster 1 and 2, Figures 2d–e, Figure S2). This can be exemplified. by the SA-CVZ volcanic arc geological context. Here, deeply sourced springs fed by surrounding arc volcanism carry waters with high arsenic concentrations (often >1 mg/L, Supplementary Table 2) derived from arc geothermal inputs^23^, along with several metals (*e.g*., Sb, Cu) mobilized by magmatic fluids^24^. Despite the toxicity of these metals, the resident microbial community in the hot springs of the SA-CVZ volcanic arc is metabolically rich^25,26^. This is in line with our metagenomic surveys of microbial BGC potential in volcanic arcs that exhibit broad putative diversity (Figure S2). Similarly in other volcanic arc examples within our dataset, such as the AAVP, geothermal fields also produce acidic, sulfur-rich emissions with significant heavy metal enrichment in Cu, Zn, Hg, and Co^27^. Accordingly, the microbial communities from volcanic arc springs reveal significantly higher NRPS prevalence compared with all other sites (Figure 4), suggesting prolific metallophore production and metal-microbe interactions in this environment^28^.

**Figure 5-:**
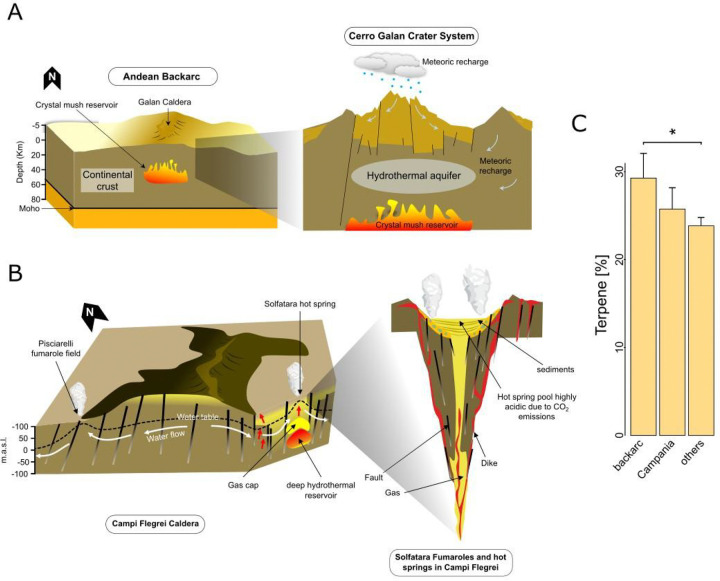
Schematic representation of backarc and post-subduction extensional volcanic systems in active plate tectonic settings and their influence on the microbial biosynthetic potential. (a) Schematic representation of the Galán system in the SA-CVZ (South American Central Volcanic Zone) backarc and its influence on the local microbial biosynthetic potential. The Galán Caldera overlies a large crystal mush reservoir within the continental crust. In contrast to volcanic arcs, backarc systems experience fewer direct discharge of magmatic gases to the surface and therefore are more scarce in the primary metal-rich fluids associated with direct magmatic degassing. They are majorly controlled by the hydrothermal circulation of deep hydrothermal reservoirs, receiving less direct input from the subducting slab, and hence their fluid chemistry is comparatively restricted. Accordingly the BGC potential diversity is lower and specialised in terpenes, likely involved in membrane stabilization. Geological and regional scale cartoons were adapted from ^118^. (b) Schematic representation of Campi Flegrei Caldera (post-subduction extensional arc), in the Campania region, Italy; “m.a.s.l.” refers to meters above sea level. At the macro scale, Campi Flegrei is influenced by a deep hydrothermal reservoir within the continental crust. The system is high in volatiles such as CO_2_ and H_2_S. These conditions support microbial communities adapted to high acidity, elevated CO_2_, and sulfide concentrations. Cross sections adapted from ^119^. (c) At the microbiological level, the predicted BGC potential in backarc systems and Campania is potentially high in terpenes, which may aid in stress tolerance, cellular protection, and adaptation to volatile-rich environments. The bar plots show the per-sample proportional representation of of terpene biosynthetic potential, computed as the number of terpene gene cluster families (GCFs) detected in each sample divided by the total number of detected GCFs in that sample (expressed as %), comparing 34 backarc samples, versus 17 Campania samples and the remaining 161 samples within active plate tectonic settings. Group differences in terpene proportions among the three tectonic settings were first assessed using a non-parametric Kruskal–Wallis test. When significant, pairwise differences were evaluated using Dunn’s post hoc test with Holm correction for multiple comparisons. Significance was coded as adjusted p < 0.05 (*).

In comparison to volcanic arcs, geothermal sites in backarc and post-subduction extensional volcanics show a contraction in putative BGC diversity. This might likely reflect differences in geological context that shape metal availability. In the SA-CVZ backarc, slab-derived inputs are less pronounced^29^, and the degree of mantle melting and subduction influence is lower than the adjacent volcanic arc^30^. Backarc fluids also interact more extensively with the overriding crust during their ascent, leading to longer residence times^31^. Unlike arcs, they lack abundant direct conduits for magmatic gases to reach the surface, resulting in diminished fluxes of the metal-rich fluids typical of direct slab inputs^31^. By contrast, volcanic arc conduits provide more rapid and direct routes for fluid ascent, delivering a broader mixture of magmatic and hydrothermal fluids^31,32^. The greater fluid diversity in the arc might correspond with the broader predicted GCF diversity we observe, whereas the putative metagenomic GCF profile of backarc sites is accordingly less diverse (Figure S2), and potentially specialized in terpenes compared to other active plate tectonic sites (Figure 5).

While the biosynthesis of some terpene compounds relies on metal cofactors such as Zn, for isopentenyl diphosphate isomerase^33^, and Fe–S clusters for methylerythritol phosphate^34^, their high occurrence in backarc systems could suggest that these pathways might be preferentially maintained for functions beyond metal metabolism. In particular, terpenes can serve versatile roles in membrane fortification^35,36^ and protection against fluctuating pH^37,38^, helping microbial communities withstand CO_2_^−^ and H_2_S-rich, metal-poor conditions^39–41^. A comparable trend is observed in the system of the Campi Flegrei, in the Campania region in Italy, where shallow CO_2_ degassing can reach up to 4000–5000 tones per day^42^, and the Solfatara crater and surrounding fumaroles (*e.g.*, Pisciarelli) continuously emit CO_2_-rich, sulfurous fumes^42,43^. Despite such geochemical harshness, cultivation efforts have yielded spore-forming isolates (*e.g.*, *Alicyclobacillus mali* from Pisciarelli mud) whose genomes encode enzymes for unusual isoprenoid terpene biosynthesis^44^. It is worth highlighting this cultivation evidence aligned with our metagenomic data from these Campania systems, which similarly reveals high predicted terpene GCF occurrence (Figure 5). Both the SA-CVZ backarc and Italian Campania cases illustrate that when volcanic systems skew toward high volatile (CO_2_ and H_2_S) flux and away from direct input of metal-rich magmatic fluids, microbial secondary metabolism may shift towards a greater representation of terpene-associated putative GCFs.

### Potential microbial strategies for metal homeostasis and competition in Mid-Ocean Ridge Ecosystems

Tectonic settings at spreading centers, such as at mid-ocean ridges (MORs), exhibit biosynthetic profiles distinct from both volcanic arcs and backarc systems. In the RVB, geothermal fields like Gunnuhver lie directly atop the Mid-Atlantic Ridge, tapping basaltic magmas and mantle-derived volatiles. At Gunnuhver, subsurface temperatures reach ~290 °C, with localized zones exceeding 300 °C^45^. Previous studies of (plume influenced) Icelandic solfataric fields have reported enrichment in RiPPs, such as archaeocins^18^. Consistent with these observations, RVB hot spring microbiomes in our dataset showed the highest representation of RiPP- and NRPS-associated gene cluster families (Figure S8), which together accounted for 64% of detected GCF occurrences within this group. The widespread occurrence of predicted RiPP-associated GCFs suggests that antimicrobial production may play an important role in these communities: many thermophiles produce heat-stable bacteriocins and related peptides that help maintain competitiveness within dense biofilm assemblages coating sinter deposits in hot springs^47,48^. The elevated presence of putative NRPS-associated GCFs might reflect a dual adaptation to the environment of MOR hot springs where basalt-hosted fluids are indeed enriched in metals such as Fe, Cu, Zn, and various trace elements, primarily due to intense fluid-rock interaction and magmatic degassing at depth^49,50^. However, the bioavailability of these metals is highly variable, largely because of rapid mineral precipitation, which removes metals from the fluid phase as the fluids ascend and cool^50,51^. In this context, NRPS-associated metallophore pathways may serve a dual role: scavenging essential trace nutrients under limitation^9,10^, and detoxifying excess metals when concentrations become inhibitory^52,53^. The observed putative GCF composition thus could represent a strategy shaped by MOR geochemistry, which creates a complex system for metal bioavailability^49,50,54^.

Overall, our findings suggest that tectonic setting is associated with distinct patterns in microbial biosynthetic GCF composition across geothermal environments. Volcanic arc systems show higher representation of NRPS-associated GCFs, consistent with geochemical conditions influenced by magmatic inputs, whereas back-arc environments, particularly those at higher elevation, are characterized by comparatively lower GCF diversity and increased representation of terpene-associated families. In divergent settings such as mid-ocean ridges, GCF profiles dominated by NRPS- and RiPP-associated families might align with environmental conditions linked to metal availability and dense microbial communities. By influencing magmatic differentiation^55^, pressure of mineral stabilization^56,57^, and fluid-rock interaction pathways^58^, the local tectonic context indirectly influences the chemical and thermal conditions that subsurface microbial communities experience in geothermally influenced ecosystems.

### Microbial terpenes as potential biosignatures in modern and ancient geothermal systems

**Figure 6 -:**
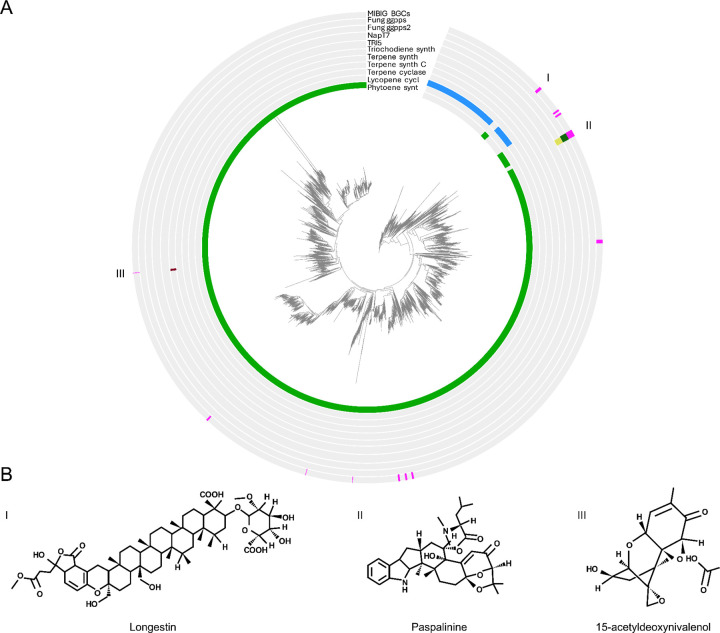
Phylogenetic diversity of terpene biosynthetic core enzymes and representative terpene chemistries. (a) Maximum-likelihood phylogeny of predicted terpene biosynthetic core proteins identified in geothermal metagenomes together with experimentally characterized reference sequences from the MIBiG (Minimum Information about a Biosynthetic Gene) database. Concentric rings indicate annotation features associated with terpene biosynthesis, including MIBiG reference clusters and functional domains identified by antiSMASH (e.g., terpene synthases, terpene cyclases, phytoene synthases). Labeled regions (I–III) highlight clades containing reference enzymes used to illustrate representative terpene chemical scaffolds. (b) Representative metabolites produced by experimentally characterized terpene biosynthetic pathways corresponding to the highlighted phylogenetic regions. These compounds (e.g., Longestin, Paspalinine, and 15-acetyldeoxynivalenol) illustrate known terpene structural diversity associated with nearby reference enzymes in the tree and provide biochemical context for the broader terpene diversity observed in geothermal microbial communities. Such compounds and related terpene scaffolds may represent candidate molecular biomarkers for microbial activity in geothermal environments.

While our analysis focuses on broad-scale patterns in putative GCF composition, phylogenetic reconstruction of terpene biosynthetic core proteins revealed extensive predicted diversity across geothermal sites and tectonic settings. Placement of metagenome-derived sequences alongside reference terpene synthases provides an overview of the putative terpene biosynthetic diversity present in these systems (Figure 6), many of which may hold potential as molecular indicators of past life and environmental conditions^59^. Although most biomarker applications to-date rely on plant-derived terpenes (e.g., triterpenoids) preserved in sedimentary archives from the mid-Paleozoic onwards^60,61^, recent work has highlighted the presence of microbial terpenes in modern and ancient hydrothermal deposits^59,62^.

Fossilized microbial lipids, including terpenes, serve as molecular fossils (biomarkers) that show the history of microbial life on Earth^62,63^. For example, carotenoids (a subset of terpenes) in ancient sediments are used to infer the presence and ecological roles of phototrophic microbes in past environments^64^. The structural diversity and recurrence of putative microbial terpene BGCs in our geothermal samples reinforces the view that these compounds could serve as biomarkers not only in modern geothermal environments but also in ancient settings where microbial biomass dominated, as already suggested by ^35,59^. As very early Earth environments were often geothermal^65,66^, the rich terpene diversity present today in these systems may reflect a long-standing association between geothermal activity and microbial terpene biosynthesis^67–69^. It is also worth remarking that the biosynthetic pathways for terpenoids are equally ancient and widespread among microorganisms^70,71^. If preserved in the rock record, microbial terpenes extend the application beyond the plant-derived molecules typically used, broadening both the range of environments and the geological time periods that can be reconstructed^59^.

Among our sites, the SKVP stands out as particularly relevant for the study of palaeoenvironments and the preservation of microbial biosynthetic signatures on Earth and potentially beyond. These hydrothermal systems are linked to Late Cenozoic intraplate volcanism driven by mantle plume–lithosphere interaction, which generated widespread volcanic centers and elevated regional heat flow^72,73^. Unlike hydrothermal systems at convergent margins, the SKVP is hosted in a tectonically inactive, paleo convergent, intracontinental plume setting but still shows evidence of long-lived geothermal activity and elevated regional heat flow^74^. Geothermal springs in the region are largely fed by meteoric waters, and there is minimal evidence for the contribution of magmatic gases or metals, which contrasts with present-day subduction-related hydrothermal systems where such inputs diversify the chemistry of the fluids^75,76^.

**Figure 7-:**
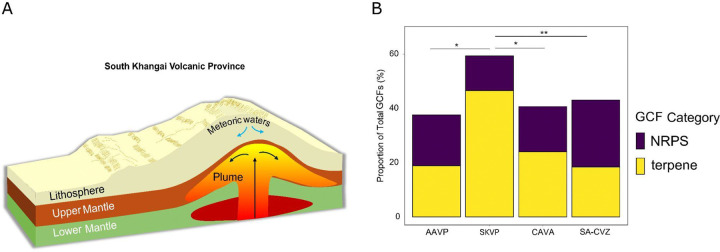
Geological context and distribution of biosynthetic gene cluster families (GCFs) across the South Khangai Volcanic Province (SKVP). (a) Conceptual cross-section of the plume–lithosphere interaction in the SKVP, modified from ^72^, where meteoric waters circulate through the lithosphere sitting above a mantle plume. (b) Proportions of NRPS (nonribosomal peptide synthetase) and terpene GCFs across different volcanic provinces: AAVP (Aeolian Arc Volcanic Province, n=10), SKVP (n=7), CAVA (Central American Volcanic Arc, n=88), and SA-CVZ volcanic arc (South American Central Volcanic Zone, n=46). Differences in NRPS-to-terpene ratios among volcanic provinces were assessed using a Kruskal–Wallis test. When significant, pairwise differences were evaluated using Dunn’s post hoc test with Holm correction for multiple comparisons. Significance is coded as p < 0.05 (*), p < 0.01 (**).

Continental plume volcanism has also been hypothesised in other planetary bodies^77–79,^ and directly inferred on Mars^78^. These systems could offer prolonged heat sources that might have potentially sustained subsurface hydrothermal habitats even as surface waters waned^80–82^. The SKVP plume-heated, meteoric-fed springs (Figure 7) thus might provide a terrestrial analogue for such environments, emerging as an intraplate-hosted simple hydrothermal circuit, driven by volcanic heat but with limited magmatic metal flux.

Interestingly, our analysis of the SKVP geothermal sites revealed the elevated presence of putative terpene-associated GCFs (47%), a value nearly double that observed in tectonically active convergent margins at volcanic arcs, where terpene contributions never exceeded 25% (Figure 7b, S8). High terpene occurrence and volatile organic compounds have also been documented in the Outokumpu deep drill site in Finland, which is hosted within a paleoserpentinized ultramafic complex, emplaced and metamorphosed during the Svecofennian orogeny^83^. The recurrence of terpene-dominated signatures in inactive plate tectonic locations either plume-related (*e.g* SKVP), or ancient intraplate settings (*e.g* Outokumpu) might suggest that long-lived hydrothermal circulation with limited magmatic metal inputs could potentially foster modern microbial communities that preferentially invest in membrane-protective and stress-mitigating terpene pathways. Such parallels point to the potential persistence of terpene biosynthetic strategies in hydrothermal systems over geological timescales, as terpene signatures could be constantly present even if plate tectonics ceases.

The elevated presence of predicted terpene-associated GCFs in the SKVP plume-related geothermal systems may highlight a functional contrast with the active convergent margin tectonics of volcanic arcs, where NRPS pathways are more frequent (Figure 7b). In arc settings such as the SA-CVZ, CAVA, and AAVP, hydrothermal fluids are directly influenced by magmatic degassing^84^, and subducting slab metal input^85^. Under these conditions, NRPS systems could provide a selective advantage by producing metal-binding metabolites that mitigate toxicity^86,87^. Similar high occurrence of NRPS-associated families is observed in other tectonically active volcanic settings, such as the Kolumbo submarine volcano (Santorini)^88^, shallow-water hydrothermal vents in the Azores^89^, and the Barren Islands volcanic arc in Southeast Asia^90^. In contrast, the SKVP hot springs are primarily fed by meteoric waters circulating through the continental crust, with limited magmatic input but constant heat-flux from remnants of the intraplate plume. Here, other environmental factors, such as the local thermal regime and the arid, high altitude desert-like nature of the region, might favor the protective and stabilizing functions of terpenes^35,91^. These compounds are well-suited to mitigate oxidative stress^92,93^, maintain membrane integrity under variable temperature and pH conditions^94–96^, and enhance UV protection^97^. Thus, intraplate inactive tectonic systems hosting residual geothermal activity may have the potential to foster microbial communities that harbor terpene-focused strategies of stress resilience, in contrast to the NRPS-associated metal response typical of volcanic arcs in active convergent margins of subduction zones.

## Conclusion

**Figure 8-:**
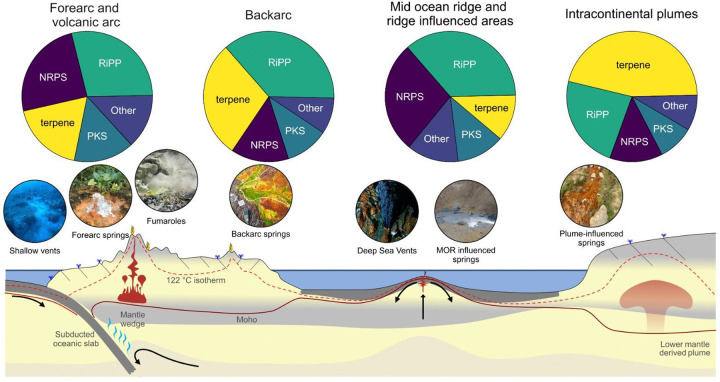
Schematic cross-section of representative tectonic regimes and associated geothermal systems in this study, illustrating large-scale geodynamics potentially influencing microbial biosynthetic gene cluster family diversity. In volcanic arcs, fluids sourced from subducting slabs and magmatic degassing might support NRPS-linked (nonribosomal peptide synthetases) pathways for metallophore production. Back-arc springs, with reduced direct magmatic input, could shift toward stress-adaptive terpene pathways. Mid-ocean ridge (MOR) systems, influenced by divergent tectonics and mantle plumes, might display NRPS- and RiPP-linked (ribosomally synthesized and post-translationally modified peptides) repertoires reflecting potential metal-microbe interactions and microbial competition. Intraplate plume-influenced settings might be characterized by high presence of terpene GCFs potentially reflecting scarce direct magmatic input in this system. GCF charts represent the relative proportions of GCF categories within the SA-CVZ (South American Central Volcanic Zone) volcanic arc, SA-CVZ backarc, RVB (Reykjanes Volcanic Belt) and SKVP (South Khangai Volcanic Province) respectively. Moho and 122°C isotherm are representative general changes across tectonic settings. Cross section adapted from ^98^.

Understanding how geological and geochemical gradients relate to microbial biosynthetic potential across planetary scales provides insight into the ecological organization of microbial communities in extreme environments. Here, we show that large-scale geological context could be associated with distinct patterns in biosynthetic gene cluster family GCF composition across globally distributed geothermal systems. These results suggest that tectonic and geochemical settings provide an important, yet previously underexplored, framework for interpreting variation in microbial biosynthetic potential (Figure 8). Expanding comparative metagenomic surveys across diverse geological environments will further clarify how Earth system processes correlate with microbial functional diversity and may help identify environments enriched in specific biosynthetic capacities relevant for future bioprospecting efforts. More broadly, linking microbial functional potential with planetary processes contributes to emerging biosignature frameworks aimed at reconstructing past environments on Earth and guiding exploration of potentially habitable, tectonically active worlds beyond our planet.

## Methods

### Sample acquisition and locations

The present work encompasses sampling campaigns spanning different tectonics settings distributed worldwide (Supplementary Methods, Supplementary Table 3), and following a large-scale sampling approach described in ^98^ (Figure 1). Environmental metadata and geological context were integrated based on the extended geochemical and tectonic framework outlined in ^98^, which emphasizes the role of deep subsurface inputs in structuring microbial communities. This broader context allowed us to systematically categorize geothermal sites by their underlying tectonic regime (backarc, volcanic arc, forearc, outer forearc and on axis ridges), facilitating standardized comparisons across disparate geographic regions.

Briefly, at each site, the primary thermal feature (i.e., venting point) was identified based on temperature, pH and conductivity gradients in combination with visual cues (e.g., gas bubbling, mineral precipitates), as described by ^98^. Microbial cells were retrieved from fluids collected directly from the venting orifice using a titanium pipe to avoid air and metal contamination, sampled through a silicone tubing attached to a portable peristaltic pump, and filtered through Sterivex 0.22 μm cartridges (MilliporeSigma). At least 2 L of hydrothermal fluid was filtered at each site and filters stored at –20 °C on-site, except for the SA-CVZ volcanic arc and backarc where samples were transported in liquid nitrogen dry shippers. Sediment samples were collected in close proximity (within centimeters) to the fluid vents using sterile, acid-washed PTFE spatulas and transferred into 50 mL centrifuge (i.e., Falcon) tubes. Sediment aliquots were also sampled for subsequent analysis of trace elements concentration and CNS content in each site. Approximately 40–45 mL of sediment was transferred into 50 mL centrifuge tubes. Collected sediment samples were immediately frozen at –20 °C and kept at this temperature during transport to the laboratory, where they were stored until further analyses. Gas-phase and water samples for dissolved gases were collected in pre-evacuated 250 mL Giggenbach bottles containing 50 mL of 4 N NaOH, following the method of ^99^, to minimize atmospheric contamination. Additionally, copper tubes were used to sample noble gases at each site, as detailed by ^99^.

Physicochemical parameters of the fluids were measured *in situ* using a thermocouple and a multiparameter probe (HANNA, HI98196). The thermocouple was used to record pool temperatures directly at the main vent inlet. Freshly venting fluids were analyzed with the multiprobe, which measured pH, oxidation-reduction potential, specific conductivity, dissolved oxygen, and total suspended solids. For fluid temperatures exceeding 55 °C, corresponding to the operational limit of the dissolved oxygen sensor, samples were cooled in closed containers prior to measurement to allow analysis within instrument specifications. Cooling was performed under conditions minimizing gas exchange with the atmosphere. The impact of this procedure was evaluated through laboratory comparisons of dissolved oxygen measurements obtained before and after controlled cooling, which indicated that deviations remained within the analytical uncertainty of the sensor under our measurement conditions.

The dataset analyzed in this study comprised 219 metagenomic assemblies. In addition to assemblies generated within this project (n = 216), three publicly available metagenomic assemblies from the Guaymas Basin were retrieved from the NCBI Sequence Read Archive to improve representation of geothermal systems from underrepresented tectonic settings. These samples were selected based on (i) comparable environmental context (hydrothermal/geothermal sediments), and (ii) availability of raw sequencing data. Collection strategy for deep-sea hydrothermal vent microbial biomass at the East Pacific Rise (EPR; 9°50 N, 104°17 W) was described in ^100^. Briefly, a total of nine samples were recovered using both experimental microbial colonization devices deployed by the ROV Jason and the deep-submergence vehicle Alvin. Samples were collected from Crab Spa (9°50.39’ N, 104°17.48’ W: 2503-m depth), P-vent (9°50.28’ N, 104°17.47’ W: 2506-m depth), Teddy Bear (9°50.50’ N, 104°17.51’ W: 2514-m depth), Bio9 (9°50.30’ N, 104°17.30’ W: 2503-m depth), Tica (9°50.39’ N, 104°17.49’ W: 2511-m depth), Perseverance (9°50.95’ N, 104°17.59’ W: 2507-m depth).

### Geochemical analysis

The ionic composition of the geothermal fluids were investigated using ion chromatography (IC) on filtered water samples using a Metrohm ECO IC system, following the standardized procedures detailed in ^101^. In the field, fluids were filtered through 0.22 μm membrane filters and collected in acid-washed plastic containers. To prevent contamination and to ensure analytical accuracy, both filters and collection vessels were rinsed with sample fluid three times prior to use. Samples were stored at 4 °C and processed within recommended holding times depending on target analytes (Cl^−^, Br^−^, NO_3_^−^, NO_2_^−^, SO_4_^2−^, PO_4_^3−^, Ca^2+^, Na, K, Mg_2_^+^, NH_4_^+^). For chloride preservation, ethylenediamine (EDA) was added in accordance with ^102^, when applicable. Cation and anion analyses were performed in separate runs using Metrosep C 4 and A Supp 5 columns, respectively. Samples were diluted with ultrapure water (typically 1:10) to maintain conductivity below 600 μS/cm, which was critical for optimal chromatographic resolution and column longevity. Additional pre-treatment steps were applied selectively based on sample composition: silver ion exchange was used in samples with elevated chloride concentrations to reduce interference with anion detection, while solid-phase extraction (C18) was applied to samples with high dissolved organic content to improve chromatographic resolution ^101^.

Chromatographic separation was achieved using a guard column followed by an analytical column and conductivity detector. For anion analysis, chemical suppression was applied to reduce background conductivity and enhance analyte signals. Compounds were identified based on retention times matched to certified standards, and quantification was performed using multipoint calibration curves (0.1–10 mg L−^1^). Calibration linearity was assessed using the coefficient of determination (r^2^), and only curves with r^2^ ≥ 0.999 were accepted. Calibration was verified by periodic analysis of a 1 ppm multistandard every 10 samples to monitor instrument stability and peak drift ^101^.

All samples were analyzed in triplicate. Quality control included routine analysis of blanks to verify low background signal and absence of carryover, as well as standard reference solutions to assess analytical accuracy, with acceptable recoveries in the range of 80–120% ^101^. This workflow enabled robust quantification of major ions (*e.g*., sulfate, chloride, sodium, potassium, calcium, magnesium) across diverse geothermal samples.

Concentrations of trace elements were determined by inductively coupled plasma mass spectrometry (ICP–MS) following the protocols described by ^103^. Briefly, filtered and acidified hydrothermal fluid samples were analyzed on an Agilent 7900 ICP–MS equipped with a collision/reaction cell to minimize polyatomic interferences. Calibration was carried out using multi-element standards spanning 0.01–100 µg L−^1^, with internal standards (e.g., Rh, In) added to correct for instrumental drift. Accuracy and precision were verified against certified reference materials and matrix-matched standards, ensuring reliable quantification even in high-salinity geothermal fluids. The procedure allows detection of trace metals of biological relevance (e.g., Fe, Mn, Co, Ni, Cu, Mo, W, V, As) at sub-ppb levels.

Gas composition analysis was performed at the Volcano Observatory of the Universidad Nacional de Costa Rica. Headspace gases (He, H_2_, O_2_, Ar, N_2_, CH_4_) were analyzed using an Agilent 7890a gas chromatograph equipped with two HP-molesieve columns (Agilent 19095P-MSO) maintained at 30 °C. Methane was detected with a flame ionization detector, while the remaining gases were quantified using thermal conductivity detectors. Following gas chromatograph analysis, the NaOH solution from the Giggenbach bottles was titrated with 0.1 N HCl to determine CO_2_ content. The CO_2_/CH_4_ ratio was calculated by determining the total moles of CO_2_ and CH_4_ in each sample. Carbon isotope compositions (δ¹³C) of gas samples were measured using a Picarro G2201-I analyzer, following acidification of the NaOH solutions extracted from the Giggenbach bottles. δ¹³C values (reported in ‰ relative to the PDB standard) were calibrated against a set of eight reference standards ranging from +2.42‰ to −37.21‰, including internationally recognized standards such as NBS19 and Carrara Marble.

### DNA extraction, metagenomic sequencing and BGC analysis

DNA was extracted with a combination of the DNeasy PowerSoil Kit (QIAGEN), and a modified phenol-chloroform DNA extraction method adapted for low biomass samples ^13^. Sequencing was carried out by Novogene (UK) using the NGS DNA Library Prep Set (Cat No.PT004) and Illumina platform is NovaSeq X Plus. Before sequencing DNA samples were quantified using a Qubit (invitrogen) dsDNA Broad Range or High Sensitivity assay and visually inspected on a 1% agarose gel colored with EtBr. Briefly, genomic DNA was randomly sheared into short fragments, which were then end repaired, A-tailed, and ligated to Illumina adapters. Adapter-ligated fragments were size selected, purified, and processed according to Novogene’s standard workflow prior to sequencing. Libraries were quantified using Qubit and qPCR, and fragment size distributions were assessed prior to pooling and sequencing. Sequencing quality control and read filtering were performed by Novogene, including removal of adapter-contaminated reads, reads containing >10% ambiguous bases (N), and reads containing >50% low-quality bases (Q ≤ 5). Per-sample sequencing depth and statistics are reported in Supplementary Table 4.

Raw reads were processed using the GEOMOSAIC pipeline (github.com/giovannellilab/Geomosaic, Corso et al. 2025, *submitted*). Reads were trimmed using fastp (default parameters) and assembled with metaSPAdes (v. 3.15.5)^104^ with k-mers -k 33, 55, 77, and 127, and filtering for minimum contig length of 2000 base pairs. All the generated 219 assemblies were analyzed with antiSMASH (antibiotics and Secondary Metabolite Analysis SHell) 7.0^105^, using default settings, including KnownClusterBlast/MIBiG (Minimum Information about a Biosynthetic Gene cluster) 4.0^106^ matching and the product/substrate prediction modules to investigate the richness of biosynthetic potential in geothermal environments. AntiSMASH detects BGC classes by rule-based domain profiling and, when possible, predicts candidate product scaffolds. To account for redundancy among similar BGCs predicted across multiple assemblies, antiSMASH GenBank outputs were dereplicated into gene cluster families (GCFs) using BiG-SCAPE (Biosynthetic Gene Similarity Clustering and Prospecting Engine) 2.0^107^. BiG-SCAPE computes pairwise similarity relationships among BGCs based on domain architecture, sequence similarity, and gene organization, generating similarity networks in which nodes represent individual BGCs and edges represent similarity scores.

GCFs were defined using a similarity cutoff of 0.5 following standard BiG-SCAPE clustering parameters. Analyses were performed separately for major biosynthetic classes (NRPS, PKS, RiPPs, and terpenes) and included reference BGCs from the MIBiG (Minimum Information about a Biosynthetic Gene cluster) database^126^. Within each similarity network, connected components were interpreted as gene cluster families, thereby grouping homologous or partially fragmented BGCs originating from different assemblies into unified biosynthetic units.

### BGC dereplication and novelty assessment

Biosynthetic profiles were represented as presence–absence matrices of GCFs per metagenome. No abundance estimates were inferred and read mapping was not performed. Analyses therefore focus on biosynthetic diversity rather than quantitative abundance, to ensure comparability across samples with heterogeneous sequencing depth and assembly completeness.

A GCF was considered present in a sample when at least one member BGC belonging to that family was detected within the corresponding assembly. Because metagenomic assemblies may contain incomplete or fragmented BGCs, dereplication at the GCF level reduces redundancy by clustering partial and complete clusters sharing conserved biosynthetic architecture. Analyses were therefore conducted at the family level rather than individual BGC predictions. GCFs detected in fewer than 5% of samples were removed to reduce sparsity. Sequencing depth (log₁p-transformed read counts) was included as a conditioning variable in multivariate analyses to control for technical variation. To assess whether sequencing depth influenced biosynthetic profiling and clustering, the number of reads per metagenome (Supplementary Table 4) was used as a proxy for sequencing effort. Initial ordination analyses showed significant correlations between sequencing depth and ordination axes (Spearman ρ = −0.68, p < 2.2 × 10−^1^⁶ for Axis 1; ρ = −0.28, p = 2.3 × 10⁻⁵ for Axis 2), indicating a potential technical gradient. To control for this effect, sequencing depth was log-transformed (log1p) and included as a conditioning variable in constrained ordination models (capscale). After conditioning on sequencing depth, correlations between ordination axes and sequencing depth were no longer significant (Axis 1: ρ = −0.04, p = 0.51; Axis 2: ρ = 0.005, p = 0.94).

Results were combined into a single feature table and imported in R (v. 4.3, R Core Team 2024). The subsequent statistical analyses, data processing and plotting were carried out using the phyloseq package^127^, vegan^128^ and ggplot2^129^ packages. Relationships between environmental variables (e.g., temperature, pH, conductivity, crustal thickness) and biosynthetic composition were assessed using partial redundancy analysis (RDA) conditioned on sequencing depth, with significance evaluated using permutation tests (999 permutations). Sample clusters were identified using k-means clustering of RDA site scores (k = 3), selected based on elbow and silhouette criteria. While silhouette width was maximized at k = 2, the elbow criterion indicated diminishing returns beyond k = 3, and k = 3 provided improved ecological interpretability and separation of major geothermal system types (Supplementary Figure S9). Differences among clusters were evaluated using PERMANOVA and ANOSIM, and homogeneity of dispersion was verified using betadisper (p = 0.31), indicating that separation reflected compositional differences rather than unequal variance among clusters. Alpha diversity was calculated using Shannon’s index and compared among tectonic settings using Kruskal–Wallis tests with Benjamini–Hochberg correction.

To evaluate biosynthetic novelty, BiG-SCAPE similarity networks including MIBiG reference clusters were analyzed using NetworkX^108^ (v 1.10) in Python (v 3.14). Connected components were defined as GCFs and classified as putatively known when containing at least one MIBiG reference cluster, or putatively novel otherwise. For novelty analyses, the complete set of predicted BGCs from all metagenomes was retained, ensuring that rare or sample-specific biosynthetic gene clusters were included in the assessment. Networks were visualized in Cytoscape^109^ (v 3.10.3), then imported to Gephi (v 0.1) for optimum data visualisation. This network-based framework provides a conservative estimate of novelty by identifying biosynthetic families lacking detectable similarity to experimentally characterized clusters^107^.

### Taxonomic profiling from assembled contigs

Taxonomic composition was inferred from assembled contigs using CAT (Contig Annotation Tool)^110^ 5.10.3. CAT assigns lineages to contigs using protein-level homology searches and a lowest common ancestor framework. Genus-level assignments were retained when support exceeded 0.70. Contig lengths were extracted from assembly FASTA files and aggregated by genus within each metagenome. Taxonomic profiles were calculated as the proportion of classified assembled base pairs assigned to each genus per metagenome. Taxa present in fewer than 20% of samples were removed prior to analysis to reduce sparsity. Because assembly recovery and classification success can vary with sequencing effort, sequencing depth (log-transformed total reads) was included as a covariate and controlled in downstream multivariate analysis. Profiles were Hellinger-transformed and summarized using principal component analysis (PCA); the first ten principal components (cumulative variance explained = 44.7%) were retained for downstream analyses. Variation partitioning (RDA; adjusted R^2^) was used to quantify independent and shared contributions of environmental variables and taxonomic composition to GCF distributions while conditioning on sequencing depth.

### Phylogenetic analysis of core terpene biosynthetic proteins

To characterize putative terpene biosynthetic diversity, we performed a phylogenetic analysis of predicted core terpene biosynthetic proteins recovered from antiSMASH-annotated biosynthetic gene clusters. AntiSMASH GenBank output files (.gbk) generated from metagenomic assemblies were parsed using a custom Python script implemented with Biopython^111^ (v 1.71). Coding sequences (CDS features) were screened for terpene-associated annotations contained within the gene_functions and sec_met_domain qualifier fields produced by antiSMASH. CDS entries were retained as candidate terpene core biosynthetic genes when annotation text contained terpene-related functional keywords corresponding to terpene synthases or associated prenyltransferases (including: *phytoene_synt*, *Lycopene_cycl*, *terpene_cyclase*, *Terpene_synth*, *Terpene_synth_C*, *trichodiene_synth*, *NapT7*, *TRI5*, *fung_ggpps*, and *fung_ggpps2*).

For each retained feature, the translated amino acid sequence provided in the translation qualifier was extracted. FASTA headers encoded sequence provenance, including source GenBank file, record identifier, locus tag, and antiSMASH functional annotations. Extracted protein sequences from metagenomic assemblies were combined with reference terpene biosynthetic proteins derived from curated biosynthetic gene clusters to provide phylogenetic context.

Protein sequences were aligned using MAFFT^112^ (v7; with the flags “--auto”). Alignment trimming was performed using ClipKIT^113^ (v 2.3.0) with default parameters to remove poorly aligned positions and reduce noise while retaining phylogenetically informative sites. Maximum-likelihood phylogenetic trees were inferred using IQ-TREE 2^114^ under the LG+G4+F amino acid substitution model. Branch support was evaluated using 1,000 ultrafast bootstrap replicates and 1,000 SH-like approximate likelihood ratio tests (SH-aLRT). Phylogenetic tree display and annotation was performed through iTOL^115^ v7. The whole pipeline was run on the HPC IBiSco (Infrastructure for BIg data and Scientific COmputing; project PON R&I 2014–2020 dell’ Avviso 424/2018 - Azione II. 1.) at the University of Naples Federico II, using SLURM^116^ (v 2.11.5).

## Supplementary Material

Supplement 1

Supplement 2

## Data Availability

Raw sequences are available through the European Nucleotide Archive (ENA) under the Umbrella Project CoEvolve PRJEB55081. The NCBI samples from EPR are available through the NCBI PRJ PRJNA1092253. The NCBI samples from the Guaymas basin are available through the NCBI PRJ number PRJNA879229. A complete shell script and jupyter notebook containing all the steps to reproduce our antiSMASH pipeline and figures in this paper is available at: github.com/giovannellilab/antismash_BGC_geothermal_env with DOI 10.5281/zenodo.15785035 along with all collected geochemical and environmental data.
