# Supplementary material for: Tectonic setting shapes microbial biosynthetic potential across global geothermal environments
Source: bioRxiv. 2026 Apr 30:2025.09.14.675129. Preprint. [Version 2] doi: 10.1101/2025.09.14.675129 (PMC13142401; doi:10.1101/2025.09.14.675129)
Supplement: Supplement 1 [file media-1.pdf]

Sample locations

Reykjanes Volcanic Belt (RVB)

We collected 10 samples from 3 geothermal spring regions in Krýsuvík (63.895451; -22.056914), Gunnuhver (63.819344; -22.68), and Hveragerdi (64.008117; -21.17949). The sampling areas were characterized by absence of vegetation, and hot soil located both to the north and south of the main vent area. The soil type was predominantly clayey/clayey-sandy.

South Khangai Volcanic Province (SKVP)

We collected a total of 7 samples from 4 geothermal springs in the regions of Övörkhangai and Bayankhongor. The Khujirt seep (46.9019233; 102.7696788) was surrounded by anthropic structures and tubings, probably placed to collect the water. The Shargaljuut spring (46.33309; 101.224945) was located near Erdenetsogt village, within the Bayankhongor Province. This was a popular area for locals, known for the hot springs with beneficial properties. The Nariinteel (46.128628; 101.605386) and Taragt (46.273908; 102.473959) springs were located in Övörkhangai in flat areas with low vegetation.

South American Central Volcanic Zone (SA-CVZ)

We collected a total of 68 samples from 50 geothermal spring locations in the SA-CVZ. The full expedition report to the SA-CVZ is present in [1]. Briefly, deeply-sourced fluids were collected in the Andean Convergent Margin, between 17 °S and 24 °S, to understand interactions between microbiology, deeply-sourced fluids, the crust, and tectonic parameters.

Central America Volcanic Arc (CAVA)

We collected a total of 88 samples from 56 geothermal spring locations in the CAVA . Full details of sampling are described in [2]. Briefly, samples were located along the southern CAVA, to investigate whether geological setting and context could influence subsurface microbiology.

Tuscan-Latium Volcanic Province (TLVP)

We collected a total of 7 samples from 7 geothermal springs in the TLVP. Sorgente di Chiorba spring (43.1527486; 10.853645) emerges from an opening located in a stone made structure located in the Biancane natural park; Sasso Pisano (43.1676069; 10.8667609) is a degassing site located close to a

camping area; Soffioni Boraciferi (43.1435272; 10.8153437) is situated within a large bubbling pool in an area with very little vegetation; Torre Alfina (42.747778; 11.948202) is a pool of meteoric water with diffuse degassing; Solfatara di Ferento (42.504311; 12.1371561) is a site of diffuse degassing within a river margin; San Cristoforo (42.3984606; 12.0592911) is a pool of hot water carved in a carbonate mound; Stagno Bianco (41.7045854; 12.5365787) is a spring in a small canyon located in a degassing area.

Campania

We collected a total of 17 samples from 12 geothermal springs in the Campania region. Bagnone (43.181228; 10.832435); Acque Cantani (41.314806; 13.891273), Terme Caracciolo Forte (40.690045; 15.251482); Capasso geyser (40.690045; 15.251482), Sorgente Ferrata (40.651557; 15.229379); Grotta dell'acqua (40.825108; 14.059695); Lido lo scoglio (40.82538; 14.077299); Madonna dei Lattani (41.302894; 13.984734); Piccolo Inferno (41.314487; 13.894006); Stufe di Nerone (40.826908; 14.076022); Sorgente Petrinum (41.125096; 13.890296); Varchera (40.705666; 15.222618).

Aeolian Arc Volcanic Province (AAVP)

We collected a total of 10 samples from 10 geothermal springs in the AAVP. Acque Calde (38.417502; 14.959262) is a pool characterized by diffused degassing located in the island of Vulcano; Fangaia Vulcano (38.416171; 14.959509) is a mud pool which has been previously geochemically characterized[3]; Levante Bay (38.4167042; 14.9599477), Geyser Vulcano (38.4175871; 14.9599809), Black Point (38.3814; 15.0618), Bottaro (38.3819; 15.0637) are shallow-water hydrothermal vents; Hotel Oasi (38.637719; 15.075066) is located inside the touristic Hotel Oasi in Panarea where hydrothermal waters come from a well; Bagno Secco Sorgente Rossa (38.491391; 14.908525) is a spring located in an area with abundant vegetation; Terme San Calogero Stufa (38.477998; 14.910223) is located in an old thermal stove in the thermal establishment San Calogero in Lipari; Casa Fulco (38.798403; 15.237873) is a water well constantly monitored by the National Volcanology and Geophysics Institute in Italy (INGV).

References

1. Bastoni, D. *et al.* Targeting deeply-sourced seeps along the Central Volcanic Zone. *Open Res. Eur.* **4**, 226 (2024).

2. Basili, M. *et al.* Subsurface microbial community structure shifts along the geological features of the Central American Volcanic Arc. *PLOS ONE* **19**, e0308756 (2024).

3. Capasso, G., Favara, R. & Inguaggiato, S. Chemical features and isotopic composition of gaseous manifestations on Vulcano Island, Aeolian Islands, Italy: An interpretative model of fluid circulation. *Geochim. Cosmochim. Acta* **61**, 3425–3440 (1997).
