# Supplementary material for: Tectonic setting shapes microbial biosynthetic potential across global geothermal environments
Source: bioRxiv. 2026 Apr 30:2025.09.14.675129. Preprint. [Version 2] doi: 10.1101/2025.09.14.675129 (PMC13142401; doi:10.1101/2025.09.14.675129)
Supplement: Supplement 2 [file NIHPP2025.09.14.675129v2-supplement-2.pdf]

bioRxiv preprint doi: https://doi.org/10.1101/2025.09.14.675129; this version posted April 30, 2026. The copyright holder for this preprint (which was not certified by peer review) is the author/funder, who has granted bioRxiv a license to display the preprint in perpetuity. It is made available under aCC-BY 4.0 International license.

Supplementary Figures

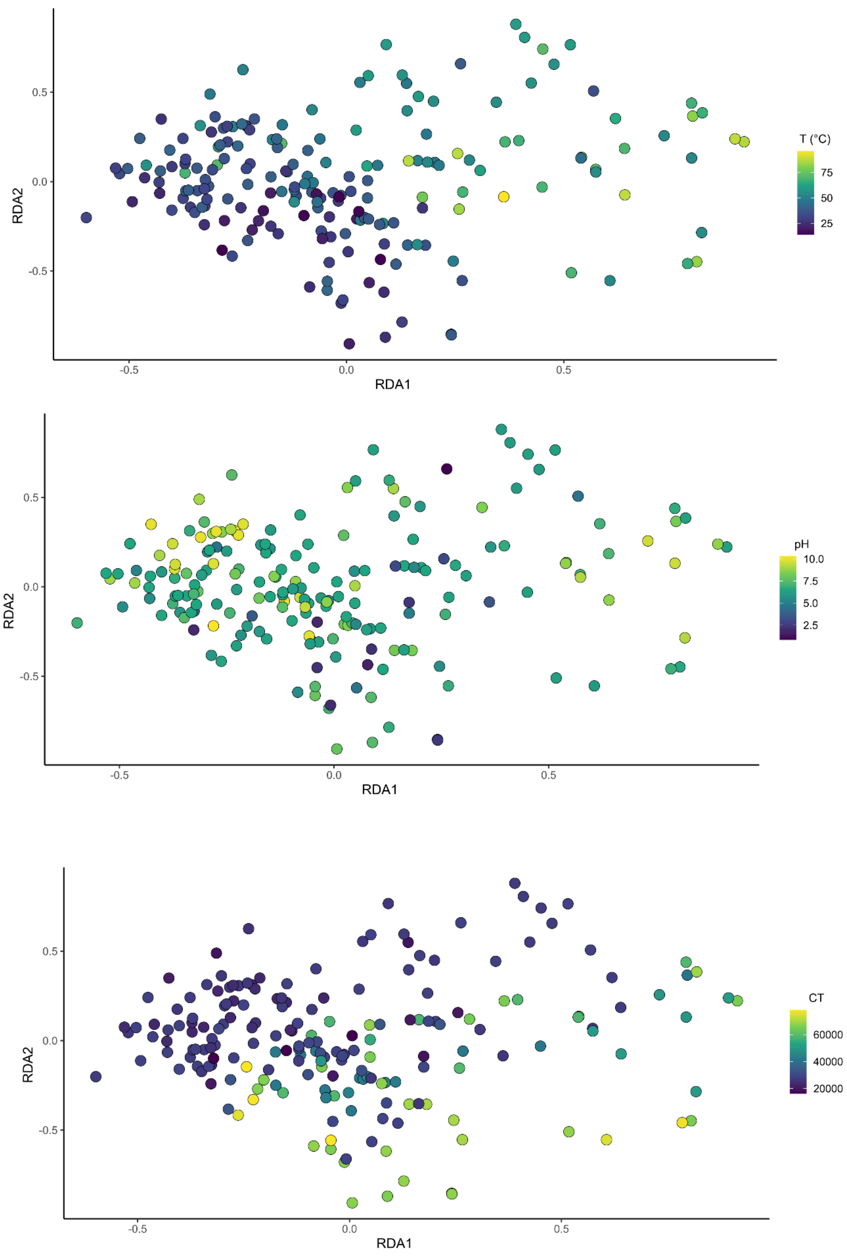

Figure S1 - Distribution of environmental gradients across geothermal samples. Top:

bioRxiv preprint doi: https://doi.org/10.1101/2025.09.14.675129; this version posted April 30, 2026. The copyright holder for this preprint (which was not certified by peer review) is the author/funder, who has granted bioRxiv a license to display the preprint in perpetuity. It is made available under aCC-BY 4.0 International license.

Redundancy analysis (RDA) showing biosynthetic gene cluster family (GCF) profiles by temperature. Middle: RDA constrained by pH. Bottom: RDA constrained by CT. Environmental variables tested for impact in the dataset using marginal PERMANOVA.

bioRxiv preprint doi: https://doi.org/10.1101/2025.09.14.675129; this version posted April 30, 2026. The copyright holder for this preprint (which was not certified by peer review) is the author/funder, who has granted bioRxiv a license to display the preprint in perpetuity. It is made available under aCC-BY 4.0 International license.

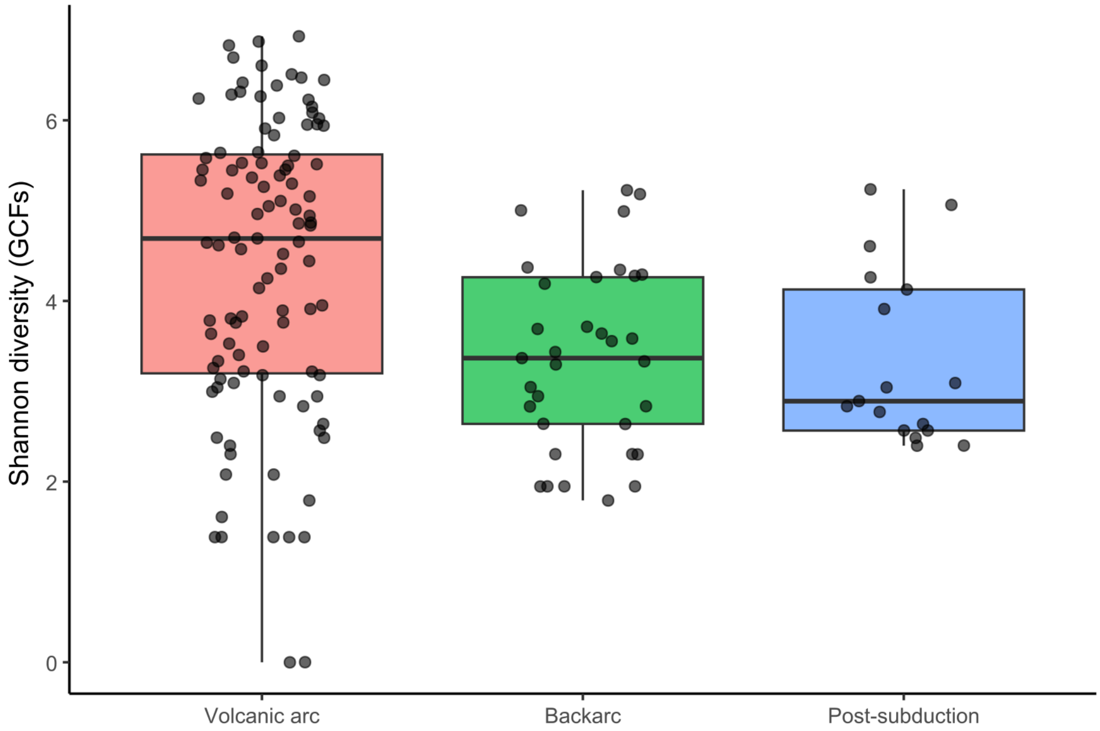

Figure S2 - Alpha diversity of biosynthetic gene cluster families (GCFs) across tectonic settings. Shannon diversity indices were calculated from presence/absence profiles of GCFs in geothermal samples grouped by tectonic context (volcanic arc, backarc, post-subduction). Diversity differed significantly among groups (Kruskal–Wallis $\chi^2 = 17.35$, $p = 1.7 \times 10^{-4}$). Volcanic arc sites ($n = 125$) showed the highest diversity (mean ± SD = 2.18 ± 0.69), significantly greater than both backarc ($n = 34$, 1.85 ± 0.57; $p = 0.0026$) and post-subduction sites ($n = 17$, 1.88 ± 0.41; $p = 0.0026$). Backarc and post-subduction sites did not differ significantly ($p = 0.99$). Boxplots display medians (horizontal line), interquartile range (box), and full range (whiskers), with individual samples shown as jittered points.

bioRxiv preprint doi: https://doi.org/10.1101/2025.09.14.675129; this version posted April 30, 2026. The copyright holder for this preprint (which was not certified by peer review) is the author/funder, who has granted bioRxiv a license to display the preprint in perpetuity. It is made available under aCC-BY 4.0 International license.

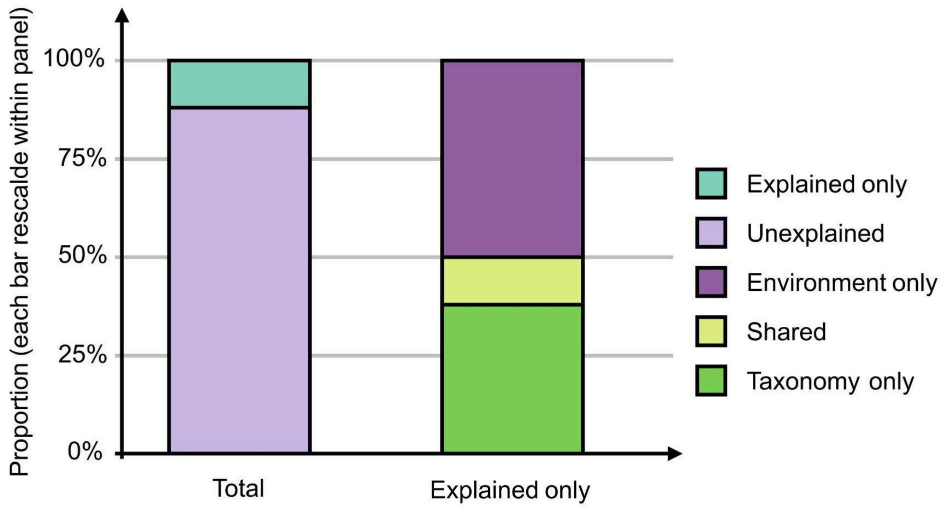

Figure S3 - Variation partitioning of GCF composition into environmental and taxonomic components. Environmental variables explain a greater proportion of variation (adjusted R^2 = 0.058) than taxonomy (adjusted R^2 = 0.046), with a smaller shared fraction (adjusted R^2 = 0.017). Together, these factors account for ~12% of total variation, with the remainder unexplained.

bioRxiv preprint doi: https://doi.org/10.1101/2025.09.14.675129; this version posted April 30, 2026. The copyright holder for this preprint (which was not certified by peer review) is the author/funder, who has granted bioRxiv a license to display the preprint in perpetuity. It is made available under aCC-BY 4.0 International license.

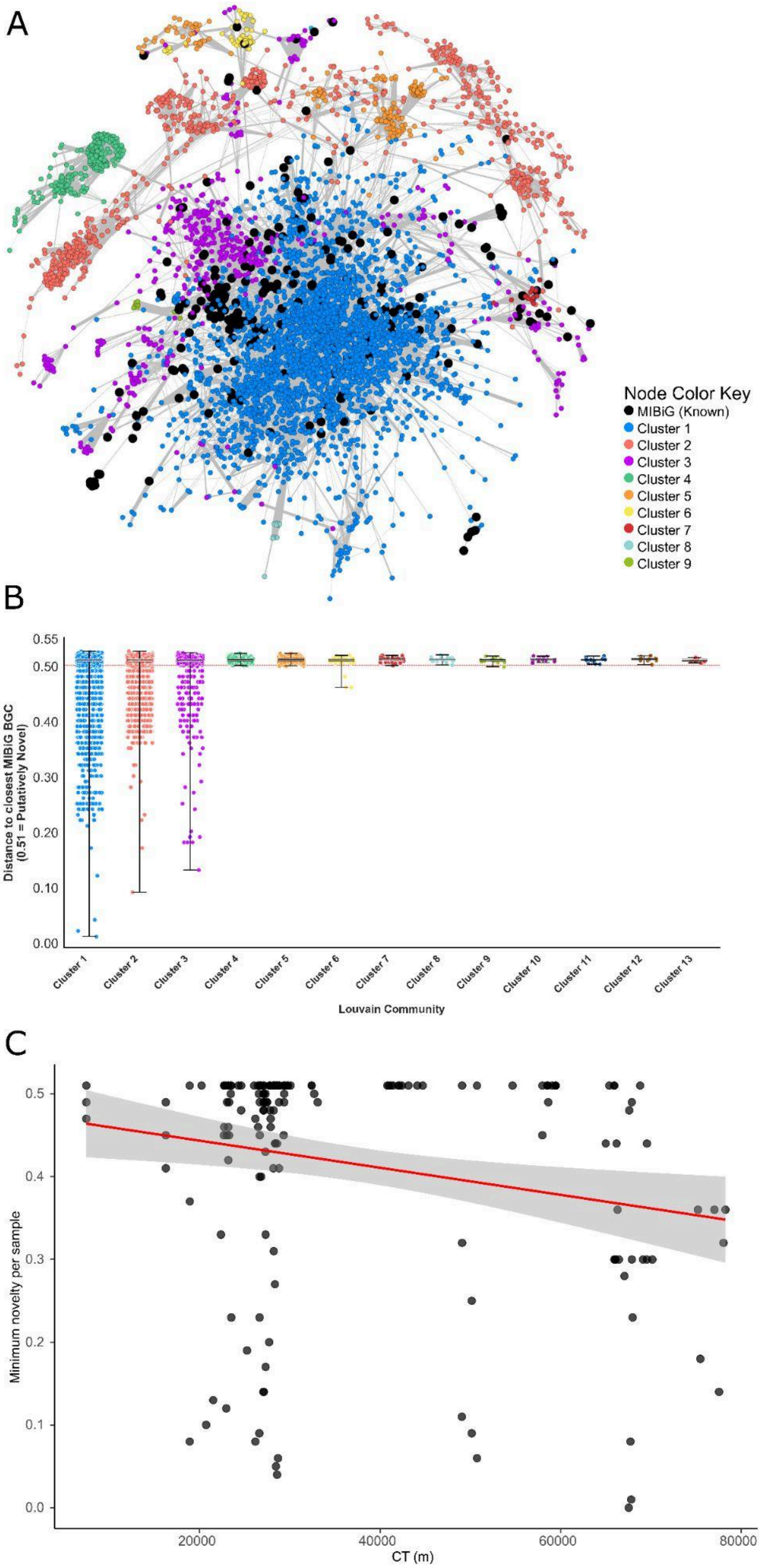

bioRxiv preprint doi: https://doi.org/10.1101/2025.09.14.675129; this version posted April 30, 2026. The copyright holder for this preprint (which was not certified by peer review) is the author/funder, who has granted bioRxiv a license to display the preprint in perpetuity. It is made available under aCC-BY 4.0 International license.

Figure S4 - Network structure and novelty of nonribosomal peptide synthetase (NRPS) biosynthetic gene clusters. (a) Similarity network of predicted NRPS biosynthetic gene clusters generated using BiG-SCAPE (Biosynthetic Gene Similarity Clustering and Prospecting Engine) at a distance cutoff of 0.5. Nodes represent BGCs and edges indicate pairwise similarity (distance $\leq$ 0.5). Nodes are colored according to Louvain communities, and experimentally characterized reference clusters from the MIBiG (Minimum Information about a Biosynthetic Gene) are shown in black. Connected components correspond to predicted gene cluster families (GCFs). (b) Distribution of distances from each putative BGC to its closest MIBiG reference cluster, grouped by Louvain community. Each point represents a single predicted BGC, with boxplots summarizing the distribution within each community. Distances of 0.51 (above the cutoff, indicated by the dashed red line at 0.5) correspond to putative BGCs with no detectable similarity to any MIBiG cluster within the threshold and are therefore considered putatively novel. Communities with higher median distances and a greater proportion of values at 0.51 represent more putatively novel NRPS BGC families. (c) Relationship between minimum NRPS BGC novelty per sample and crustal thickness (CT). Each point represents a single sample. The solid red line indicates a linear regression with 95% confidence interval (grey shading). A weak but significant negative relationship is observed (R^2 = 0.043, p = 0.0063), indicating reduced putative novelty with increasing crustal thickness.

bioRxiv preprint doi: https://doi.org/10.1101/2025.09.14.675129; this version posted April 30, 2026. The copyright holder for this preprint (which was not certified by peer review) is the author/funder, who has granted bioRxiv a license to display the preprint in perpetuity. It is made available under aCC-BY 4.0 International license.

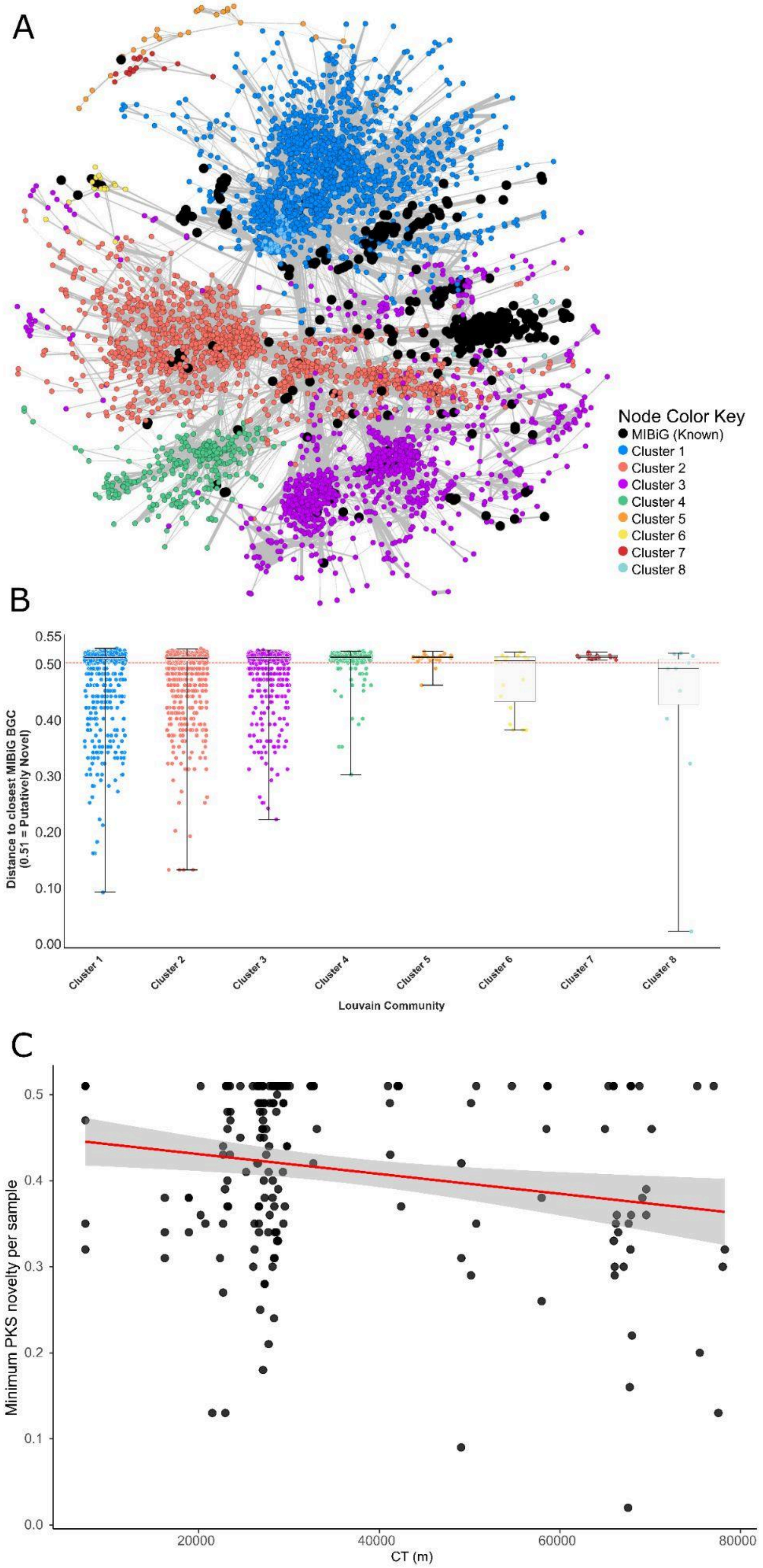

bioRxiv preprint doi: https://doi.org/10.1101/2025.09.14.675129; this version posted April 30, 2026. The copyright holder for this preprint (which was not certified by peer review) is the author/funder, who has granted bioRxiv a license to display the preprint in perpetuity. It is made available under aCC-BY 4.0 International license.

Figure S5 - Network structure and novelty of polyketide synthase (PKS) biosynthetic gene clusters. (a) Similarity network of predicted PKS biosynthetic gene clusters generated using BiG-SCAPE (Biosynthetic Gene Similarity Clustering and Prospecting Engine) at a distance cutoff of 0.5. Nodes represent BGCs (biosynthetic gene clusters) and edges indicate pairwise similarity (distance $\leq$ 0.5). Nodes are colored according to Louvain communities, and experimentally characterized reference clusters from the MIBiG (Minimum Information about a Biosynthetic Gene) are shown in black. Connected components correspond to predicted gene cluster families (GCFs). (b) Distribution of distances from each putative BGC to its closest MIBiG reference cluster, grouped by Louvain community. Each point represents a single predicted BGC, with boxplots summarizing the distribution within each community. Distances of 0.51 (above the cutoff, indicated by the dashed red line at 0.5) correspond to putative BGCs with no detectable similarity to any MIBiG cluster within the threshold and are therefore considered putatively novel. Communities with higher median distances and a greater proportion of values at 0.51 represent more putatively novel PKS BGC families. (c) Relationship between minimum putative PKS BGC novelty per sample and crustal thickness (CT). Each point represents a single sample. The solid red line indicates a linear regression with 95% confidence interval (grey shading). A weak but significant negative relationship is observed (R^2 = 0.041, p = 0.0070), indicating reduced putative novelty with increasing crustal thickness.

bioRxiv preprint doi: https://doi.org/10.1101/2025.09.14.675129; this version posted April 30, 2026. The copyright holder for this preprint (which was not certified by peer review) is the author/funder, who has granted bioRxiv a license to display the preprint in perpetuity. It is made available under aCC-BY 4.0 International license.

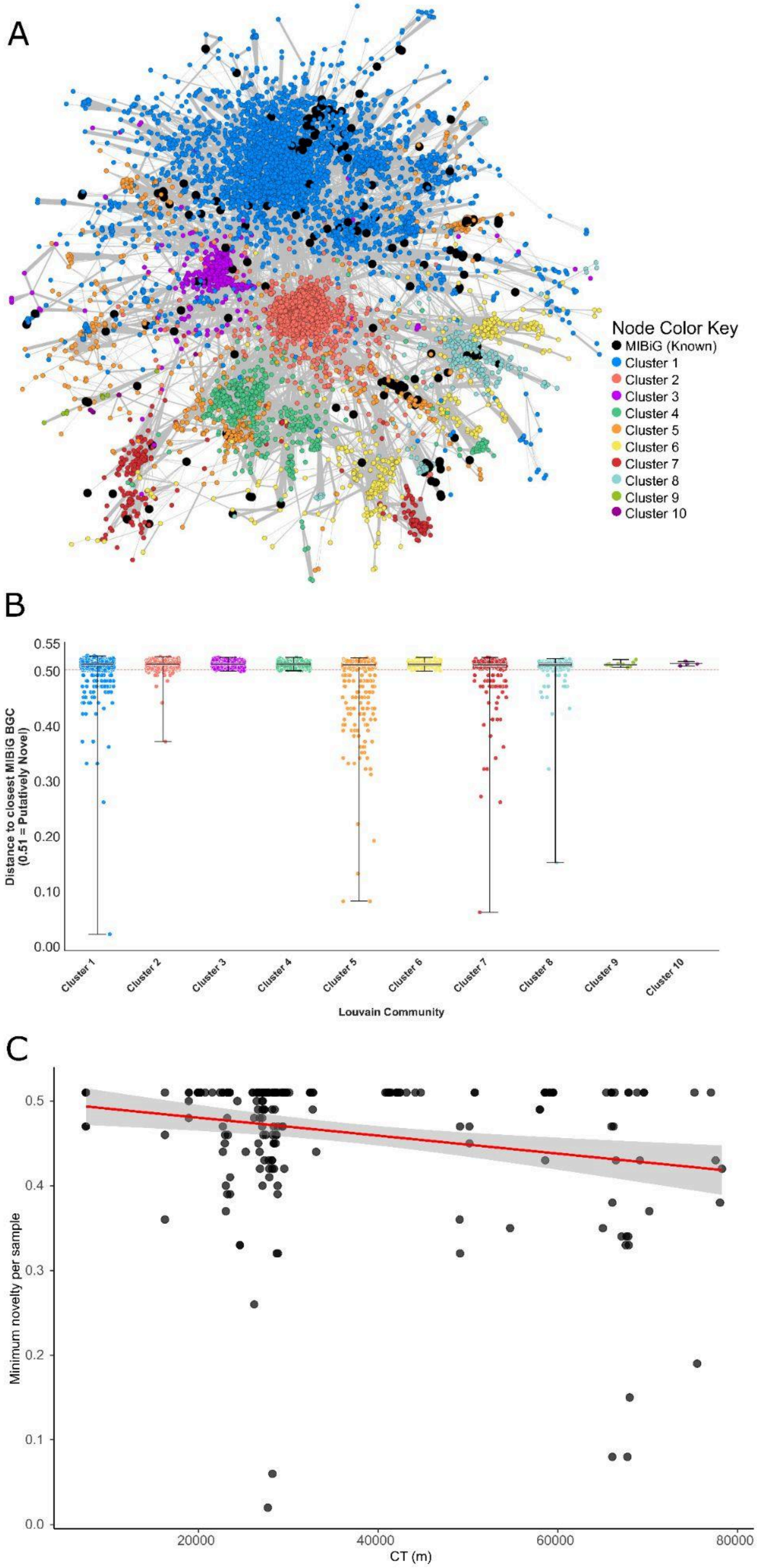

bioRxiv preprint doi: https://doi.org/10.1101/2025.09.14.675129; this version posted April 30, 2026. The copyright holder for this preprint (which was not certified by peer review) is the author/funder, who has granted bioRxiv a license to display the preprint in perpetuity. It is made available under aCC-BY 4.0 International license.

Figure S6- Network structure and novelty of ribosomally synthesized and post-translationally modified peptide (RiPP) biosynthetic gene clusters. (a) Similarity network of predicted RiPP biosynthetic gene clusters generated using BiG-SCAPE (Biosynthetic Gene Similarity Clustering and Prospecting Engine) at a distance cutoff of 0.5. Nodes represent BGCs (biosynthetic gene clusters) and edges indicate pairwise similarity (distance ≤ 0.5). Nodes are colored according to Louvain communities, and experimentally characterized reference clusters from the MIBiG (Minimum Information about a Biosynthetic Gene) are shown in black. Connected components correspond to predicted gene cluster families (GCFs). (b) Distribution of distances from each putative BGC to its closest MIBiG reference cluster, grouped by Louvain community. Each point represents a single predicted BGC, with boxplots summarizing the distribution within each community. Distances of 0.51 (above the cutoff, indicated by the dashed red line at 0.5) correspond to putative BGCs with no detectable similarity to any MIBiG cluster within the threshold and are therefore considered putatively novel. Communities with higher median distances and a greater proportion of values at 0.51 represent more putatively novel RiPP BGC families. (c) Relationship between minimum putative RiPP BGC novelty per sample and crustal thickness (CT). Each point represents a single sample. The solid red line indicates a linear regression with 95% confidence interval (grey shading). A weak but significant negative relationship is observed ($R^2 = 0.050$, $p = 0.0013$), indicating reduced putative novelty with increasing crustal thickness.

bioRxiv preprint doi: https://doi.org/10.1101/2025.09.14.675129; this version posted April 30, 2026. The copyright holder for this preprint (which was not certified by peer review) is the author/funder, who has granted bioRxiv a license to display the preprint in perpetuity. It is made available under aCC-BY 4.0 International license.

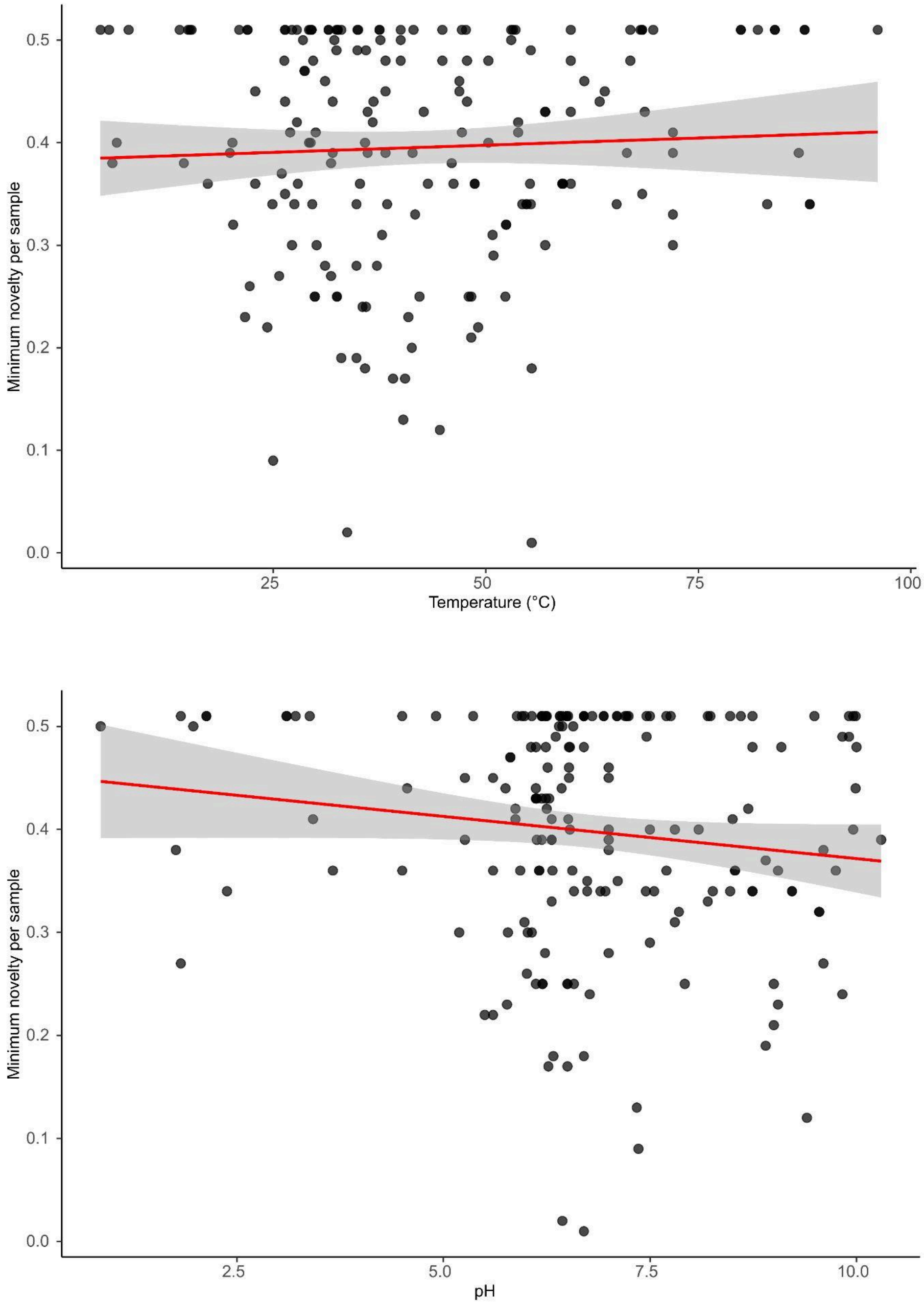

Figure S7 - Relationship between minimum terpene biosynthetic gene cluster (BGC) novelty

bioRxiv preprint doi: https://doi.org/10.1101/2025.09.14.675129; this version posted April 30, 2026. The copyright holder for this preprint (which was not certified by peer review) is the author/funder, who has granted bioRxiv a license to display the preprint in perpetuity. It is made available under aCC-BY 4.0 International license.

per sample and environmental variables. (a) Temperature and (b) pH. Each point represents the minimum distance to a reference MIBiG cluster observed within a sample. Red lines indicate linear regression fits with 95% confidence intervals (grey shading). In both cases, relationships are weak, with low explained variance and no statistically significant association (temperature: $R^2 \approx 0.002$, $p = 0.57$; pH: $R^2 \approx 0.018$, $p = 0.072$), indicating that terpene BGC novelty is largely independent of these local physicochemical gradient

bioRxiv preprint doi: https://doi.org/10.1101/2025.09.14.675129; this version posted April 30, 2026. The copyright holder for this preprint (which was not certified by peer review) is the author/funder, who has granted bioRxiv a license to display the preprint in perpetuity. It is made available under aCC-BY 4.0 International license.

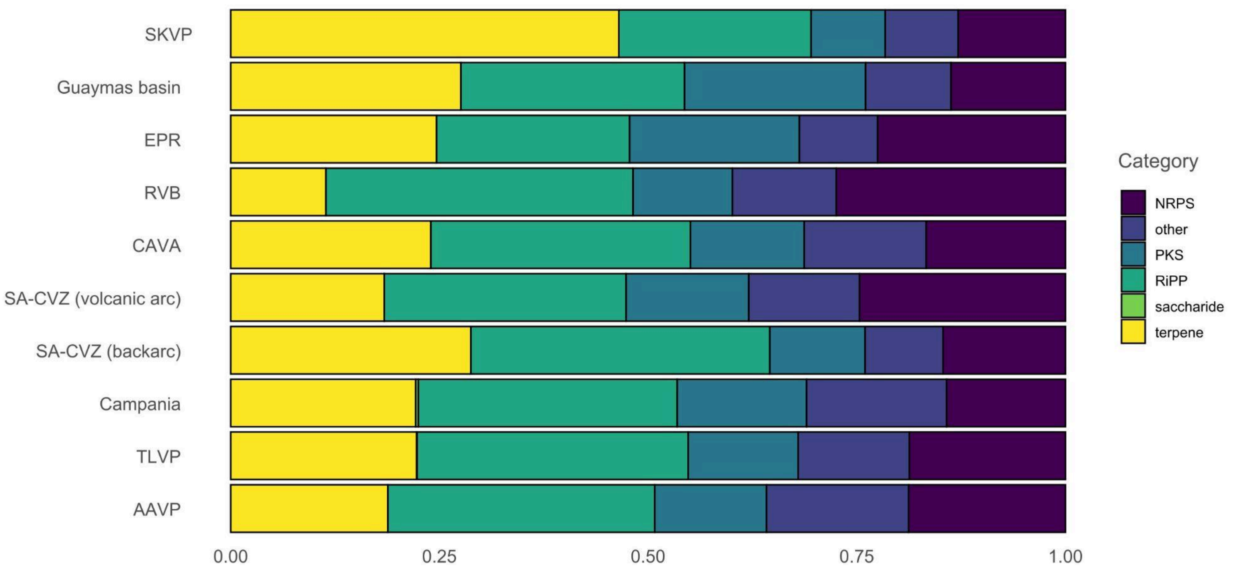

Figure S8 - Biosynthetic gene cluster family (GCF) composition across geothermal and volcanic regions. Stacked bar plots summarize the distribution of GCF categories in metagenomic assemblies from different global sites. Differences in GCF composition highlight variation in biosynthetic potential associated with distinct geological and geochemical contexts. At the volcanic province scale, the same patterns as of our Clusters are possible to be seen. SA-CVZ (South American Central Volcanic Zone) volcanic arc samples (most abundant in Cluster 2) exhibit high representation of NRPS (nonribosomal peptide synthetases) (24.7%) and RiPP (ribosomally synthesized and post-translationally modified peptides) GCFs (29.0%), whereas SA-CVZ back-arc systems (abundant in Cluster 3) exhibit increased representation of RiPPs (35.8%) and terpenes (28.7%) and reduced representation of NRPS GCFs (14.7%). . Samples from the CAVA (Central American Volcanic Arc) reveal a relatively balanced putative biosynthetic landscape. RiPPs still have higher representation (31.1%), followed by terpenes (24.0%), NRPSs (16.7%), PKSs (13.6%), and "other" clusters (14.6%). In Italy, which spans diverse tectonic regimes, from subduction-related island arcs (*e.g.*, AAVP (Aeolian Arc Volcanic Province)) to post-subduction extensional arcs (*e.g.*, Campania and TLVP (Tuscan-Latium Volcanic Province)), the GCF distribution is varied. AAVP samples show the lowest terpene contribution to BGC pool amongst Italian samples (18.8 %, while Campania and

bioRxiv preprint doi: https://doi.org/10.1101/2025.09.14.675129; this version posted April 30, 2026. The copyright holder for this preprint (which was not certified by peer review) is the author/funder, who has granted bioRxiv a license to display the preprint in perpetuity. It is made available under aCC-BY 4.0 International license.

TVLP had a contribution of 23% both), and Campania samples show a modest NRPS contribution (14% in comparison to 19% for both AAVP and TVLP). TVLP samples show a slightly elevated RiPP proportion 33%, followed by AAVP 32% and Campania 31%. PKS show small contributions in all Italian samples, reaching no more than 15% for each of them. “Other” GCFs contribute 17% for Campania and AAVP, and 13% for TVLP. Geothermal springs in the RVB (Reykjanes Volcanic Belt), located along the active Mid-Atlantic Ridge, as well as deep-sea hydrothermal vents in the Guaymas Basin and deep seeps in the SKVP (South Khangai Volcanic Province), show distinctly different biosynthetic profiles compared to all other sample sites. In the RVB (most abundant in Cluster 3), biosynthetic gene cluster families contain elevated representation of RiPPs (36.8%) and NRPSs (27.5%), while all other categories each account for less than 12%. The EPR putative GCF pool is relatively well balanced containing 24% terpene, 23% RiPP, 22% NRPS and 20% PKS, other GCFs occupy the remaining 10%. The Guaymas Basin microbiome shows the highest PKS representation across all sampled volcanic provinces (22%), terpenes were the most representative GCF category in these samples (28%), followed by RiPPs (27%) and NRPSs (14%). Other BGCs contributed modestly at 9%. SKVP samples exhibit the strongest representation of putative terpene GCFs across our global dataset (47%). RiPPs contribute 22%, NRPSs 13%, and the remaining categories together comprise the final 18%. These distributions suggest a focused yet distinct biosynthetic specialization across such geothermal systems.

bioRxiv preprint doi: https://doi.org/10.1101/2025.09.14.675129; this version posted April 30, 2026. The copyright holder for this preprint (which was not certified by peer review) is the author/funder, who has granted bioRxiv a license to display the preprint in perpetuity. It is made available under aCC-BY 4.0 International license.

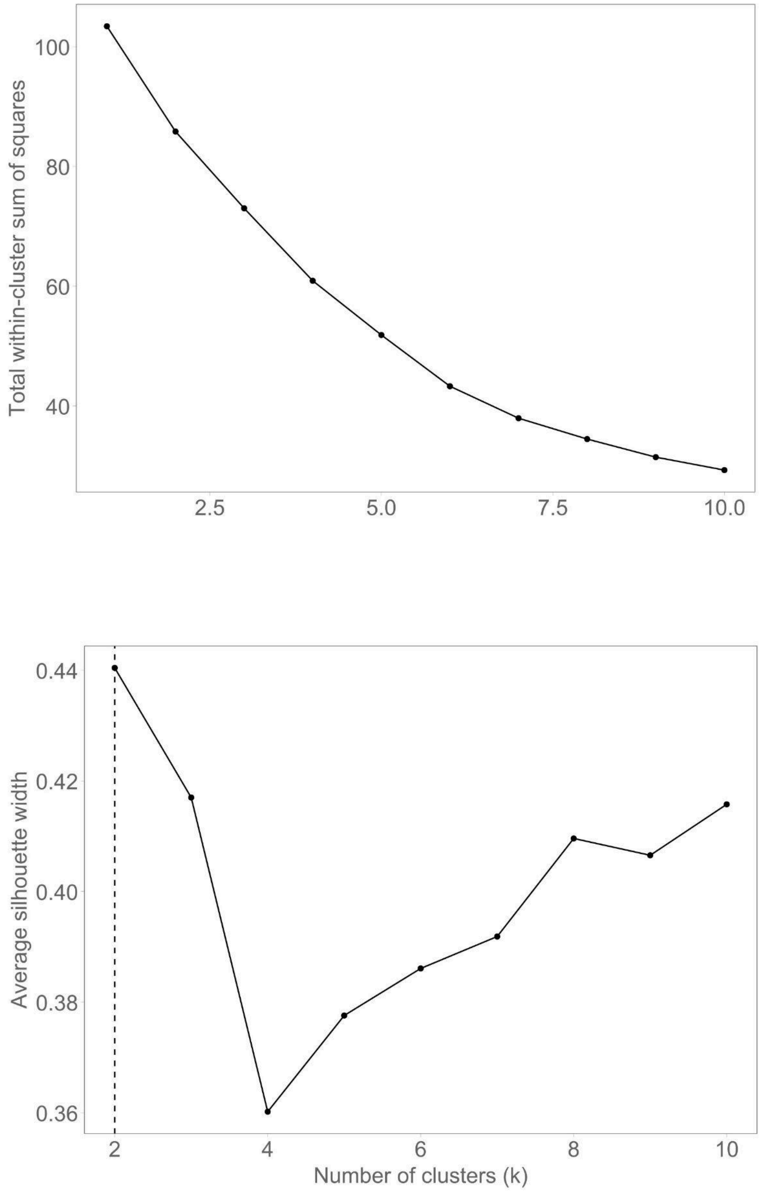

Figure S9 - Selection of the optimal number of clusters for k-means analysis. Elbow (top) and silhouette (bottom) analyses were used to evaluate clustering performance across a range of cluster numbers (k = 1–10). The elbow plot shows a progressive decrease in the within-cluster

bioRxiv preprint doi: https://doi.org/10.1101/2025.09.14.675129; this version posted April 30, 2026. The copyright holder for this preprint (which was not certified by peer review) is the author/funder, who has granted bioRxiv a license to display the preprint in perpetuity. It is made available under aCC-BY 4.0 International license.

sum of squares with diminishing returns beyond $k \approx 3$. The silhouette analysis indicates a maximum at $k = 2$, with comparable values for higher k. Based on these criteria and improved ecological interpretability, $k = 3$ was selected for downstream analyses.
